# Bipolar charge collecting structure enables overall water splitting on ferroelectric photocatalysts

**DOI:** 10.1038/s41467-022-32002-y

**Published:** 2022-07-22

**Authors:** Yong Liu, Mingjian Zhang, Zhuan Wang, Jiandong He, Jie Zhang, Sheng Ye, Xiuli Wang, Dongfeng Li, Heng Yin, Qianhong Zhu, Huanwang Jing, Yuxiang Weng, Feng Pan, Ruotian Chen, Can Li, Fengtao Fan

**Affiliations:** 1 State Key Laboratory of Catalysis, Dalian National Laboratory for Clean Energy, iChEM, Dalian Institute of Chemical Physics, Chinese Academy of Sciences, Dalian, 116023 China; 2 School of Advanced Materials, Peking University, Shenzhen Graduate School, Shenzhen, 518055 China; 3 The Laboratory of Soft Matter Physics, Beijing National Laboratory for Condensed Matter Physics, Institute of Physics Chinese Academy of Science, Beijing, 100190 China; 4 State Key Laboratory of Applied Organic Chemistry, Advanced Catalysis Center, College of Chemistry and Chemical Engineering, Lanzhou University, Lanzhou, 730000 China

**Keywords:** Chemical physics, Photocatalysis, Photocatalysis, Ferroelectrics and multiferroics

## Abstract

Ferroelectrics are considered excellent photocatalytic candidates for solar fuel production because of the unidirectional charge separation and above-gap photovoltage. Nevertheless, the performance of ferroelectric photocatalysts is often moderate. A few studies showed that these types of photocatalysts could achieve overall water splitting. This paper proposes an approach to fabricating interfacial charge-collecting nanostructures on positive and negative domains of ferroelectric, enabling water splitting in ferroelectric photocatalysts. The present study observes efficient accumulations of photogenerated electrons and holes within their thermalization length (~50 nm) around Au nanoparticles located in the positive and negative domains of a BaTiO_3_ single crystal. Photocatalytic overall water splitting is observed on a ferroelectric BaTiO_3_ single crystal after assembling oxidation and reduction cocatalysts on the positively and negatively charged Au nanoparticles, respectively. The fabrication of bipolar charge-collecting structures on ferroelectrics to achieve overall water splitting offers a way to utilize the energetic photogenerated charges in solar energy conversion.

## Abstract (web summary)

While ferroelectric materials are promising candidates for solar water splitting, most examples show poor activities. Here, authors prepare charge-collecting nanostructures on the positive and negative domains of BaTiO_3_ and demonstrate photocatalytic overall water splitting.

## Introduction

Ferroelectrics with switchable spontaneous polarization have shown tantalizing potential in memory storage and integrated microelectronics^1–5^. The power conversion efficiency exceeds unity, and a large photovoltage above the bandgap has been reported in some ferroelectrics^6–10^. The photoelectric characteristics of ferroelectrics have attracted considerable interest in solar fuel production^11–14^. Compared with the traditional charge separation driving force via drift or diffusion mechanisms in common semiconductor photocatalysts, ferroelectric semiconductors possess charge separation driving force due to spontaneous polarization^15–20^. The unique characteristics in asymmetric crystals endow ferroelectric semiconductors with the bulk photovoltaic effect (BPVE), favoring efficient photogenerated charge separation within the nonthermalizaion length^21,22^. With these photogenerated charges, the Shockley–Queisser limit for the power conversion efficiency of ferroelectric devices has been exceeded under one sun illumination (AM 1.5G)^6^. Despite the enormous potential applications, the applications of ferroelectrics in photovoltaic devices are unclear. Particularly, seldom have ferroelectric semiconductors for photocatalytic overall water splitting been reported. Although they possess a spontaneous ferroelectric polarization-induced internal field for massive charge separation and a thermodynamically suitable energy band structure for overall water splitting.

**Fig. 1:**
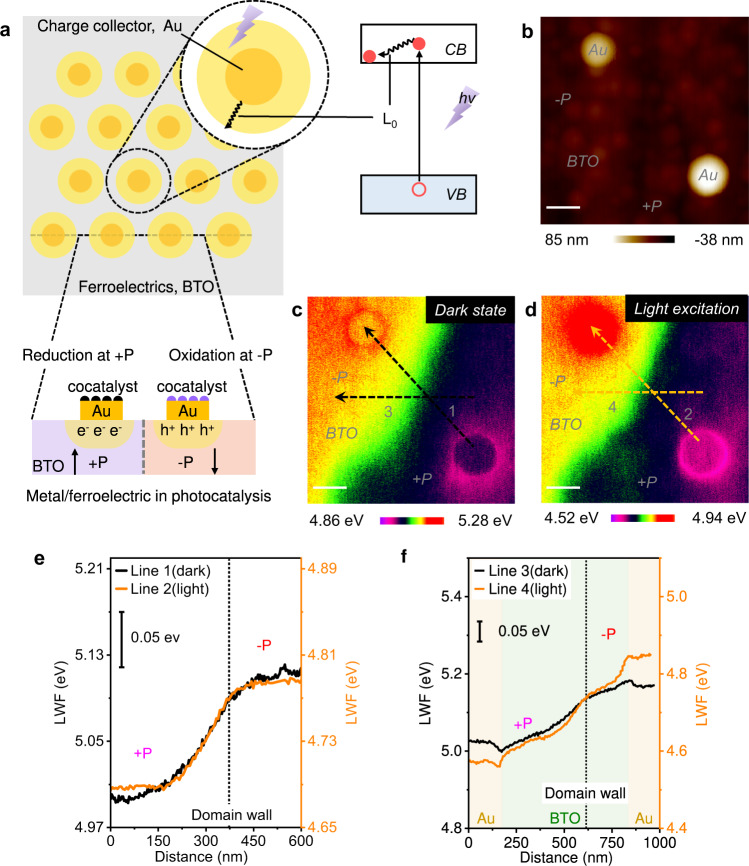
Charge separation at meta/ferroelectric interface. **a** Schematic diagram of proposed metal/ferroelectric photocatalyst. **b** AFM topography of Au particles on a BaTiO_3_ single crystal. Scale bar, 200 nm. **c** LWF of Au/BTO in the dark. Scale bar, 200 nm. **d** LWF of Au/BTO under 355 nm UV light (0.5 mW/cm^2^). Scale bar, 200 nm. **e** Line 1 (dark) and 2 (UV light) profile images were taken across two antiparallel ferroelectric domains of BTO. **f** Line 3 (dark) and 4 (UV light) profile images were taken across two antiparallel ferroelectric domains of Au/BTO.

Charge separation in ferroelectrics, known as BPVE, is often explained by two mechanisms: shift and ballistic^23^. The shift mechanism originates from a quantum phenomenon in the noncentrosymmetric crystal. This results from the coherent evolution of a quantum wave packet and the photoexcitation-induced shift of real space. The ballistic mechanism is related to the photogenerated, nonthermalized charges with asymmetric momentum distribution in the noncentrosymmetric crystal (Fig. 1a). The nonthermalized charges descend to the band bottom via a length *L*_*0*_, also called the thermalization length. *L*_*0*_ depends on materials and incident photons in tens to hundreds of nanometers. Within *L*_*0*_, all the photogenerated charges contribute to BPVE and yield the highest solar energy conversion efficiency. Hexagonal close-packed metallic electrode arrays with accurate distances were predicted to have the highest collection and utilization of photogenerated charges (Supplementary Fig. 1). Based on this principle, Spanier et al. prepare a device with a single-tip electrode contact and an array with 24 tips. The device generated a current density of 17 mA cm^−2^ under the illumination of AM 1.5 G^6^. The photogenerated charges were concentrated around every tip and were collected using an indium tin oxide (ITO) electrode. However, the fully covered ITO electrode limits the transmissivity in the ultraviolet range, where BaTiO_3_ (BTO) has the most significant absorption coefficient. As a result, the performance of this device is well below expectations. Despite the clear theoretical basis, using photogenerated charges in ferroelectrics for high-efficiency solar energy conversion remains a longstanding challenge. Thus, well-designed micro/nanostructures in ferroelectric-based semiconductors are of substantial importance in solar energy conversion. Hence, more study is needed to determine the charge separation mechanisms on the micro/nanoscale to achieve photocatalytic overall water splitting.

This paper proposes an approach to fabricate nano charge-collecting structures at the metal/ferroelectric interface to enable overall water splitting ability in a ferroelectric photocatalyst, a Au array patterned BaTiO_3_ single crystal. An anomalous concentration of photogenerated electrons and holes was found in the Au particles, located at the +P and −P domains in the BaTiO_3_ single crystal, respectively. Hence, the photogenerated charges are concentrated around the Au particle within a hemisphere of radius *L*_*0*_, a thermalization length of approximately 50 nm. The fabricated Au array/BTO photocatalysts showed substantial photocatalytic overall water splitting performance owing to the energetic photogenerated charges. The measured *L*_*0*_ is also the key experimental prescription for designing high-efficiency ferroelectrics in solar energy conversion on the nanoscales.

## Results

### Charge separation at metal/ferroelectric interface

Figure 1a presents the approach for high-efficiency solar energy conversion. In detail, Au particles in hexagonal arrays with the proper density are fabricated on the surface of a ferroelectric semiconductor substrate. An individual Au particle is an enhanced charge collection and utilization point. The cocatalysts can be deposited selectively on Au particles under illumination. The concentrated photogenerated charges within the thermalization length could promote photocatalytic activity. Conversely, the photocatalytic reduction and oxidation reactions can be separated spatially on the positive and negative polarization ferroelectric domains, respectively. In the case of photocatalytic water splitting, the hydrogen evolution reaction and oxygen evolution reaction can occur simultaneously. Solar energy conversion to chemical energy is feasible via overall water splitting.

A typical model was established to clarify the behavior of the photogenerated charges at the interface of metal and ferroelectrics. In detail, a (001)-oriented BaTiO_3_ single crystal was applied as a ferroelectric substrate, where Au nanoparticles are dispersed. The Au particles were approximately 200 nm in diameter and approximately 50 nm in thickness (Fig. 1b, Supplementary Fig. 1). Kelvin probe force microscopy (KPFM) was then applied to map the surface potential of Au/BTO under dark and light excitation conditions, as shown in Fig. 1c, d. The measured surface potential was the contact potential difference (CPD) between the Atomic force microscopy (AFM) tip and the sample. The CPD was converted to a localized work function (LWF) to gain a better understanding (Details in Methods and Supplementary Information). As shown in Fig. 1c and Line 1, the LWF of BTO at the −P and +P ferroelectric domains were markedly different. At the +P BTO, BTO exhibits downward surface band bending and a lower LWF. Conversely, BTO has an upward surface band bending at the −P domain and a higher LWF. The polarization-induced surface contrast coincides with previous results^24,25^. The LWF changes at the interface of Au/BTO are more obvious (Fig. 1c and Line 3). The LWF of BTO was even higher at the Au/BTO interface in the −P domain, indicating the formation of a Schottky-like junction with a depleting layer at the interface of Au/BTO. The oxygen vacancies in ferroelectric BTO induce n-type semiconductivity, and Au has a large work function. Similarly, at the +P Au/BTO interface, the LWF of BTO is even lower because of the formation of a quasi-Ohmic contact and an accumulation layer. The LFW of Au/BTO in the dark confirms the formation of a Schottky-like junction at −P and quasi-Ohmic contact at +P via KPFM, which is the same as previous ferroelectric devices^26–28^.

The KPFM experiments were performed under 355 nm ultraviolet (UV) light excitation (3.49 eV, ~0.5 mW/cm^2^) to investigate the photogenerated charge separation. The photon energy is higher than the bandgap of BTO (E_g_ = 3.2 eV), which is super-band illumination. The selected photon energy exceeds the bandgap of BTO but is away from the surface plasmon resonance (SPR) excitation of Au particles at this size (SPR peak position at approximately 790 nm)^29^. Thus, the plasmon resonance absorption of Au would not affect the BTO substrate at 355 nm UV illumination (Supplementary Fig. 3). As shown in Fig. 1d, under the UV light excitation, the LWF at the Au/BTO interface is changed significantly. The WLF of the domain wall shifts approximately 0.34 eV due to the BPVE of the ferroelectric polydomain in the bulk of BTO (Supplementary Fig. 7). Hence, the LWF at the domain wall between two antiparallel domains was taken as a reference^25^. The bar scales in Fig. 1c, d are 0.42 eV.

A detailed analysis of the LWF line profiles taken across the LWF images (Fig. 1c, d) is described. First, the energy band diagram of bare BTO was analyzed. As shown in Fig. 1e, lines 1 and 2 are the LWF line profiles between the two antiparallel domains in the dark and under UV light excitation, respectively. The contrast of LWF between the two antiparallel domains decreased from approximately 0.12 eV to approximately 0.1 eV. This result confirms that the ferroelectric polarization-induced downward band bending at the +P domain and reduced upward band bending at the −P domain due to the photogenerated charges transferring to the surface. The measured domain contrast is much lower than the ideal value because of screening charges^24,30^. Compared with bare BTO, the LWF line profiles across the Au particles between different BTO domains were analyzed. Figure 1f presents the LWF values extracted across lines 3 and 4 (Fig. 1f). Interestingly, under illumination, the LWF difference between the two Au particles was increased from approximately 0.18 eV to approximately 0.28 eV. This suggests that the built-in voltage of the Au/BTO interface at either the +P or −P domain was enhanced further. In contrast, the built-in voltage at the space charge regions (SCRs), such as bare BTO and common metal/semiconductor Schottky junction, decreased consistently under illumination^31,32^. The enhanced built-in voltage at the two types of Au/BTO interface shows that the Schottky-like depleting layer at the −P domain is depleted further, and the quasi-Ohmic-like accumulation layer at the +P domain is accumulated further. The above results provided strong evidence that the photogenerated charges are concentrated around the Au particles in the SCRs, agreeing with Spanier’s speculation^6^. In the surface SCRs of bare BTO and common semiconductors, the built-in voltage decreases under illumination (Supplementary Figs. 4, 5, 6)^31–34^. This phenomenon at Au/BTO is quite anomalous, entirely different from common metal/semiconductor junctions.

### Quantitative analysis of thermalization length

**Fig. 2:**
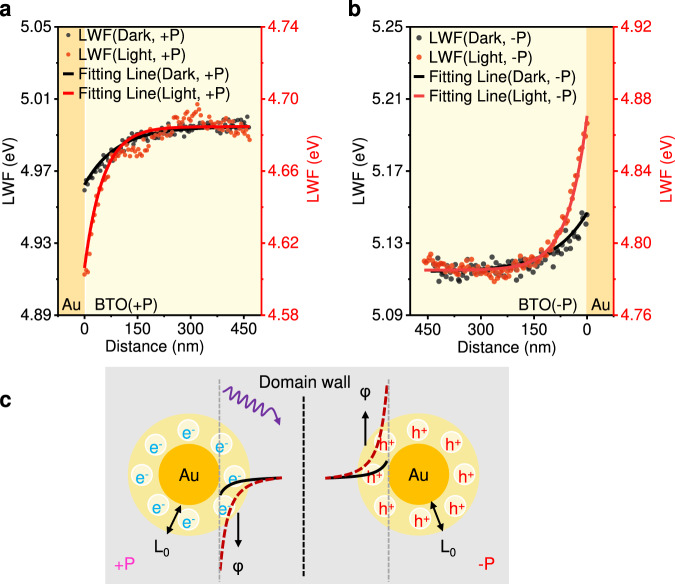
Quantitative analysis of thermalization length at Au/BTO interface. **a** LWF and fitting line at the interface of +P Au/BTO in the dark (black) and 355 nm UV light (red). In dark, φ_bi_ = 32.2 mV, L = 90.7 nm. In light, φ_bi_ = 78.5 mV, *L*_*0*_ = 52.3 nm. **b** LWF and fitting line at the interface of −P Au/BTO in the dark (black) and 355 nm UV light (red). In dark, φ_bi_ = 32.9 mV, L = 98.5 nm. In light, φ_bi_ = 85.6 mV, *L*_*0*_ = 55.1 nm. **c** Diagram of charge separation of Au/BTO at +P(left) and −P(right) in the dark (black solid line) and light (red dashed line).

Detail quantitative analysis was carried out to obtain further information. As shown in Fig. 2, the LWF at the Au/BTO interface was fitted nonlinearly to an exponential decay formula (details in Supplementary Information), giving the built-in voltage ${\varphi }_{{bi}}$ and SCR width L. At +P Au/BTO, the additional built-in voltage ${\varphi }_{{bi}}$ at the Au/BTO interface increased from 32.2 mV in the dark to 78.5 mV under UV light excitation. The ${\varphi }_{{bi}}$ is the additional built-in voltage of BTO, obtained by subtracting the surface potential of bare BTO from that of BTO at Au/BTO (Supplementary Fig. 2). Therefore, the measured ${\varphi }_{{bi}}$ is smaller than the real built-in voltage at Au/BTO heterojunction. The additional SCR width decreased from 90.7 nm to 52.3 nm in UV light. The photogenerated electrons were concentrated around the Au particles with a narrower hemisphere of radius *L*_*0*_. The thermalization length or the mean free path for a hot photoexcited electron or hole can be expressed as

1 $${L}_{0}={g}_{31}{e}^{-1}\hslash\omega /\left({\Phi {{\xi }}}^{{ex}}\right)$$

For bulk BTO single crystal, *g*_31_ $=$ 3 × 10^−9^ cm V^−1^, where $\hbar \omega$ is the incident photon energy (3.49 eV (355 nm) in this experiment); Ф is the quantum yield; *ξ*^*ex*^ is the photoexcitation asymmetry parameter; the max value of *ξ*^*ex*^ is 10^−2^–10^−3^. Most of the light is absorbed in the crystal because ${\alpha }_{{BTO}}$ ≈ 5–10 cm^–1^ at $\hbar \omega$ = 3.06 eV. Therefore, *L*_*0*_ is 10–100 nm^23^. Moreover, the excitation asymmetry parameter decreases with increasing the photon frequency. The previous experiment showed that the *L*_*0*_ = 100 nm when ħω = 3.06 eV^6^. Hence, the 50 nm *L*_*0*_ measured at 3.49 eV is reasonable. The measured *L*_*0*_ is an essential prescription in designing ferroelectric photovoltaic devices and photocatalysts.

The electric field intensity at the Au/BTO interface increased from approximately 3 kV/cm to approximately 15 kV/cm, which is five times larger (Supplementary Fig. 8). 15 kV/cm is approximately one order of magnitude higher than that of common SCRs^19,35–39^. Supplementary Fig. 8b presents the analogous phenomenon at −P Au/BTO. The steady-state charge density at the interface increased approximately 2–3 orders of magnitude under illumination (Supplementary Fig. 8c).

The concentration of photogenerated charges can be attributed to two factors, the enhanced electric field around the Au particles and the oxygen vacancies in BTO (Supplementary Fig. 10, 25-29). An intense field is concentrated approximately 150 nm away from the margin of the Au particles owing to the constructed metal–ferroelectric junctions. Under light excitation, the impact ionization of oxygen vacancies occurs within the enhanced electric field, as reported elsewhere^6,40^. A photon produces the first pair of electron e_1_ and hole h_1_ from the oxygen vacancy. In this situation, e_1_ with high mobility relaxes and produces a second pair of electron e_2_ and hole h_2_. As a result, the photogenerated charges are concentrated at the SCRs beneath BTO within a hemisphere of radius *L*_*0*_ = 50 nm around Au.

### In situ photodeposition experiments

In situ photodeposition was performed to confirm the charge transfer between ferroelectric BTO to Au. AFM topography, high-resolution scanning electron microscopy (HRSEM)/high-resolution transmission electron microscopy (HRTEM), and KPFM were applied to confirm the deposition site. Two typical photodeposition reactions based on reduction (with photogenerated electrons) and oxidation (with photogenerated holes) reactions were carried out under 355 nm UV light excitation:

$$2{{CrO}}_{4}^{2-}+5{H}_{2}O+6{e}^{-}\to {{Cr}}_{2}{O}_{3}\downarrow +10{{OH}}^{-}$$

$${{Mn}}^{2+}+x{H}_{2}O+\left(2x-2\right){h}^{+}\to {{MnO}}_{x}\downarrow +2x{H}^{+}$$

**Fig. 3:**
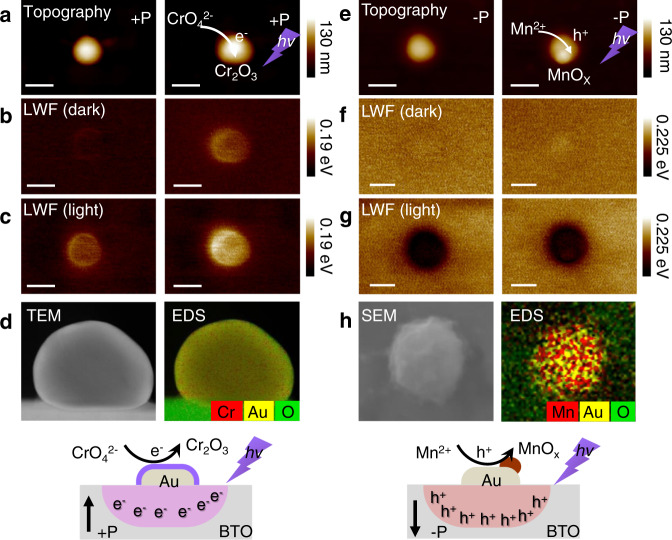
In situ photodeposition experiments. **a**–**d** Photoreduction on Au at +P BTO. **a** AFM topography of Au/BTO at +P before (left) and after (right) photodeposition. **b** LWF in the dark before (left) and after (right) photodeposition. The darker region indicates a higher LWF. **c** LWF in the light before (left) and after (right) photodeposition. Scale bar, 200 nm. **d** TEM image (left), DES image (right), and schematic illustrations of photoreduced Cr_2_O_3_ on Au at +P (bottom). **e**–**h** Photooxidation on Au at −P BTO. **e** AFM topography of Au/BTO at −P before (left) and after (right) photodeposition. **f** LWF in the dark before (left) and after (right) photodeposition. **g** LWF in the light before (left) and after (right) photodeposition. Scale bar, 200 nm. **h** SEM image (left), DES image (right), and schematic illustrations of photooxidized MnO_x_ on Au at −P (bottom).

The AFM topography in Fig. 3a shows that CrO_4_^2−^ is reduced primarily on Au particles to form a thin layer at the +P domain, indicating the formation of a Cr_2_O_3_ layer^41^. The charge density of electrons on Au particles is higher than that of BTO. Therefore, the CrO_4_^2−^ is reduced primarily to Cr_2_O_3_ on Au particles instead of ferroelectric BTO. Furthermore, the KPFM images before and after photodeposition were measured, as shown in Fig. 3b, c. After deposition, the LWF of the Au particle showed brighter contrast because of the deposited Cr_2_O_3_. The built-in voltage at Au/BTO was enhanced by the deposition of Cr_2_O_3_ under illumination. The photogenerated electrons remained concentrated around Au particles. HRTEM and energy dispersive spectrometry (EDS) mapping in Fig. 3d also showed uniformly photodeposited Cr_2_O_3_ on the surface of the Au particles. In contrast, Mn^2+^ prefers to be oxidized to MnO_x_ on the Au particle under UV light, as shown in Fig. 3e. At the same time, the KPFM images in Fig. 3f, g indicated that the charge separation at the Au/BTO interface remains the same after photodeposition. HRSEM and EDS mapping in Fig. 3h showed that MnO_x_ prefers to deposit on Au particles. Successive photodeposition in Supplementary Fig. 30 also confirmed the spatially separated charge separation. The in situ photodeposition experiments validated the proposed model in which the photogenerated electrons and holes were collected separately by the Au particles in the +P and −P domains of BTO.

### Fabricating metal/ferroelectric photocatalysts

**Fig. 4:**
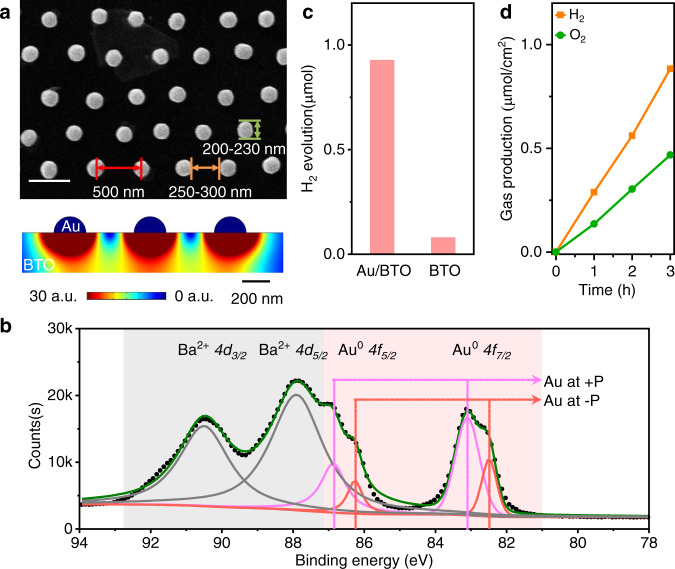
Au arrays/BTO ferroelectric photocatalysts. **a** SEM image of an Au array on a BTO single crystal and simulated electric field intensity distribution of Au array/BTO. **b** High-resolution XPS profiles of Au array/BTO. **c** Hydrogen evolution reaction of Au array/BTO and BTO. **d** Overall water splitting reactions of Au array/BTO with cocatalysts in pure water.

A ferroelectric photocatalyst was then designed based on the above experiment phenomenon and measured essential experimental prescription. Except for the thermalization length of BTO, several other factors should also be considered, such as the array density and Au particle size. The work function, metal/ferroelectric interface, and SPR of Au particles are size–dependent^42,43^. Despite the decreased density of the Au array with Au particle size, large Au particles with higher charge capacity, better metal/ferroelectric interface, and red-shift SPR are preferred. In addition, the electric field around the charged Au particle arrays should also be considered. Thus, the distance between the margin of two adjacent Au particles should be more than twice *L*_*0*_ because of the electrostatic repulsion between them (Supplementary Figs. 18, 19, 20, and 23). Based on these aspects, Fig. 4a presents appropriately designed ferroelectric photocatalysts. Periodic hexagonal close-packed (hcp) Au particles on BTO were prepared with a self-assembled polystyrene microsphere template (details in Supporting Information, Supplementary Figs. 12–16). The Au particles were approximately 200–230 nm in diameter with 500 nm periodicity. The distance between the margin of the two adjacent Au particles is approximately 250–300 nm. The electric field simulation indicated that the electric field surrounding the Au array was enhanced considerably and expanded radially but was different from the individual one. The enhanced field around the centre Au particle was almost a hemisphere and contracted horizontally compared with the individual one due to the electrostatic repulsion between the neighbor Au particles. The enhanced field extended approximately 80 nm from the margin of the Au particle. A nonenhanced area was also found between the two Au particles because of electrostatic repulsion. When the periodicity was increased to 700 nm, the enhanced electric field around the Au particle remained almost the same (Supplementary Fig. 20). When the distance between the margin of two adjacent Au particles was 100 nm, i.e., 2 × *L*_*0*_, the periodicity decreased to 300 nm. The strong electrostatic repulsion between the neighboring Au particles enables a shrunken and reduced electric field (Supplementary Fig. 19). The electric field extends less than *L*_*0*_ and cannot conform to the demand of charge collection within *L*_*0*_. The simulation results show that the designed Au array on BTO is rational.

X-ray photoelectron spectroscopy (XPS) was conducted to investigate the interfacial contact between the Au array and BTO. As shown in Fig. 4b, several peaks are layered together and can be figured as 4d peaks of Ba^2+^ and *4f* peaks of Au^0^. These peaks can fit certain constraints, such as area ratio and characterized peak location (details in Supplementary Information). Both the Au^0^ *4f*_*7/2*_ and Au^0^ *4f*_*5/2*_ peaks were divided into two peaks. The binding energy (BE) between the two peaks was approximately 0.6 eV. The difference in BE between the Au^0^ and Au^1+^ is usually approximately 1.5 eV, and it is the same with Au^1+^ and Au^3+^. Therefore, Au was in the chemical state of Au^0^, but it had a BE difference due to the constructed heterojunction with BTO. The higher BE of Au^0^ *4f* can be assigned to the Au particle at +P with quasi-Ohmic contact. The lower BE of Au^0^ *4f* can be assigned to the Au particle at −P with Schottky contact. Specifically, the BE difference of the Au particles is essential to enhancing the electric field at the Au/BTO interface. Both XPS (Fig. 4b and Supplementary Fig. 24) and KPFM confirmed the well-fabricated metal/ferroelectric heterojunctions and the polydomain ferroelectric structure of the BTO crystal. The reduction and oxidation reactions can be achieved simultaneously and separated spatially on the surface of the BTO crystal.

The photocatalytic reactions in Fig. 4c show that the Au/BTO exhibits significantly higher activity than the bare BTO, providing further experimental verification of the collection and utilization of photogenerated charges at the metal/ferroelectric interface. Water splitting was achieved in pure water after selective photodeposition (Rh/Cr_2_O_3_ and CoOOH)^44,45^ (Fig. 4d and Supplementary Fig. 23). Although perovskite BTO shows a thermodynamically suitable energy band and massive charge separation driving force for water splitting, the overall water splitting via BTO was challenging. Water splitting in pure water can be achieved by constructing nanostructures to collect and utilize photogenerated charges. These results emphasize the significance of utilizing the photogenerated charges in ferroelectrics within the thermalization length.

## Discussion

In this work, photocatalytic water splitting can be achieved in ferroelectric photocatalysts by collecting and utilizing the photogenerated charges within the thermalization length in a prototype of Au/BTO photocatalysis. KPFM reveales the concentration of photogenerated charges within the thermalization length of BTO at the Au/BTO interface. The measured thermalization length is an essential experimental prescription for fabricating high-efficiency photocatalytic and photovoltaic devices on the nanoscale. With this structure design, constructed ferroelectric photocatalysts can perform photocatalytic overall water splitting. The experimental design opens a paradigm for designing ferroelectric photocatalysts for efficient solar energy conversion.

## Methods

### Preparation of Au/BaTiO_3_

The BaTiO_3_ substrate (commercially sourced, Shanghai Daheng Optics and Fine Mechanics Co., Ltd., 5 × 5 × 0.5 mm, un-doped) was rinsed with acetone, ethanol, and deionized water by sonication for 30 min, followed by drying at 80 °C for 30 min in an oven. Next, the substrate was immersed in 1% HF solution for 1 min, thoroughly rinsed with deionized water, and dried. Then Au particles (commercially sourced, Beijing Dk Nano technology Co., Ltd., 0.1 mg/mL, 150–200 nm in diameter and 40–60 nm in thickness) were dispersed on BaTiO_3_ single crystal. Plasmon resonance absorption of Au particle is near 790 nm. Plasmon resonance absorption of Au would not affect BTO substrate at 355 nm UV illumination. Then Au/BTO samples are treated by anneal at 500 ^o^C at atmosphere for 5 h to remove the surfactant EDTA, called as Au/BTO. And then cool to room temperature naturally.

### Preparation of Au array/BaTiO_3_

Schematic of the experiments is shown in Supplementary Fig. 12. Periodic Au particles arrays are synthesized with polystyrene (PS) colloidal (Beijing Dk Nano technology Co., Ltd., 5 %) monolayers as templates. As showed in Supplementary Fig. 12, colloidal monolayer with 300/500/700 nm diameter is fabricated via a gas−liquid interfacial self-assembling strategy. Corresponding Au arrays/BTO samples are named as Au/BTO300 (300 nm spacing), Au/BTO500 (500 nm spacing), Au/BTO700 (700 nm spacing) and Au/BTOR (random spacing). Firstly, the BaTiO_3_ single crystal substrates are cleaned with acetone, ethanol and deionized water, sequentially, followed by washing with plasma cleaning for 5 min. After these processes, the surface of the BTO is sufficiently hydrophilic and ready for subsequent processes. The PS microsphere suspensions and ethanol are well mixed at a volume radio of 1:2 and then dispersed with ultrasound in ice water. Next, by self-assembly at the gas−liquid interface, monolayer PS colloidal arrays on a large area are obtained and then transferred onto BTO substrate. A thin Au film is then deposited on the colloidal monolayers and then sequentially annealed at 1000 °C for 2 h in air. PS microspheres are fully decomposed and removed from the array. The Au film is transformed into hcp Au nanoparticles in situ. The Au thickness is varied with different PS size to ensure that the Au particles are in the similar size. The commercial ferroelectric BTO single crystal is tetragonal and (001)-orientated at room temperature. The BTO is cubic and paraelectric when the temperature is higher than 120 ^o^C. During the annealing process at 1000 ^o^C, the PS microspheres are removed and BTO crystal is paraelectric. When cooling to room temperature, the BTO crystal returns to tetragonal and ferroelectric polydomain structure. The surface is consisted of random positive domain or negative domain. Therefore, the different +P Au/BTO and −P Au/BTO are formed.

### Preparation of Au random/BaTiO_3_

A control sample is prepared with random Au particle, named Au/BTOR. No PS microspheres are applied. And the Au film thickness or amount is the same as Au/BTO500. Other processes are the same as the preparation of Au arrays/BTO.

### Kelvin probe force microscopy (KPFM)

The surface potential (CPD) images and signals of the samples were measured using Kelvin probe force microscopy (KPFM; Bruker) under ambient atmosphere in the amplitude-modulated (AM-KPFM) mode. During the measurement of the surface potential, the lift mode was used with a lift height of 20 nm. 20 nm is the best choice when we take both signal noise ratio (SNR) and the signal intensity into consideration at the same time (Supplementary Fig. 11). In the lift mode, the topography and the surface potential signals were sequentially recorded. The Pt/Ir-coated Si tip was used as a Kelvin tip with a spring constant of 1–5 N/m and resonant frequency of 60–100 kHz. To measure the surface potential under illumination, 355 nm lasers (Cobolt Zouk, 99% linearly polarized) equipped with beam expansion were used. The light intensity could be adjusted from 0 to 1 mW/cm^2^. In Supplementary Fig. 7, the polarization direction of light is rotated via a half-wave plate.

### Calibration of workfunction

CPD distribution measured from freshly peeled off highly oriented pyrolytic graphite (HOPG) and fitted Gaussian distribution. The workfunction of HOPG is ranging from 4.6–5.0 eV in air via KPFM^46–49^. And thus, the workfunction of HOPG is assumed to be about 4.8 eV (Supplementary Fig. 9). Measured CPD of HOPG is of Gaussian distribution. The full width at half maximum value is about 16 mV. The measured

2 $${{{{{\rm{CPD}}}}}}=({{{{{{\rm{\varphi }}}}}}}_{{{{{{\rm{tip}}}}}}}-{{{{{\rm{\varphi }}}}}}{{{{{\rm{HOPG}}}}}})/{{{{{\rm{e}}}}}}$$

And thus, the workfunction of the tip is about 4.95 eV. In this work, we use workfunction for better understanding. The absolute value of workfunction is not accurate. The contrast between the +P and −P is more meaningful.

### Fitting of LWF with exponential decay formula

The LWF of BTO is well fitted with an exponential decay formula

3 $${LWF}={{LWF}}_{1}+{e* \varphi }_{{bi}}* {Exp}(-{L}_{1}/L)$$

The interface of BTO and gold particles is defined as the null point. *LWF* is the measured value of BTO; *LWF*_*1*_ is the LWF of BTO far away from Au, independent of Au/BTO junction’s effect; *φ*_*bi*_ is built-in voltage of BTO at Au/BTO interface; *L*_*1*_ is variate of distance away from null point; *L* is a distance constant. *L* is the distance at which the barrier height is reduced to *1/e* ≈ 0.367879441 times its built-in voltage *φ*_*bi*_. Here, we define *L* as the space charge region width. So, we can give quantitative analysis. Under illumination, the L can be defined as *L*_*0*_, the thermalization length.

### X-ray photoelectron spectroscopy (XPS)

XPS was carried out on freshly prepared samples with a Thermo ESCALAB 250Xi spectrometer using a monochromatic Al Kα source (1486.6 eV) at 15 kV and 10.8 mA. The binding energies were calibrated in relation to the C1s peak at 284.6 eV. The area ratio for Ba^2+^ *4d*_*3/2*_ and Ba^2+^ *4d*_*5/2*_ is 2:3. The area ratio for Au^0^ *4f*_*5/2*_ and Au^0^ *4d*_*7/2*_ is 3:4.

### Electronic field simulation

We simulated the electronic field distribution of Au/BaTiO_3_ nanostructures using a commercial COMSOL software. In the calculation, the Au particles is in radius of 100 nm, in the form of a hemisphere.

### Scanning electron microscope

The morphology of samples was examined by scanning electron microscopy (SEM) taken with a Quanta 200 FEG scanning electron microscope. The operation voltage was 30 kV. The morphology of Cr_2_O_3_ and MnO_x_ are examined by high-resolution scanning electron microscopy (HRSEM) taken with JSM-7900F in GBSH-S (GENTLEBEAM™ Super High resolution Stage bias mode) mode. The operation voltage was 2 kV.

### Photocatalytic water splitting experiments

The photocatalytic reactions were carried out in a Pyrex top-irradiation-type reaction vessel connected to a closed gas circulation system. Normally, a wafer of photocatalyst was immersed in 100 mL reaction solution. In photocatalytic HER, 20% CH_3_OH is added as sacrificial agent. In overall water splitting reactions, RhCrOx and CoOx were deposited on Au/BTO according to previous work^44^. And, the reaction solution for photocatalytic overall water splitting is pure aqueous solution. The system was evacuated for 60 min to ensure complete air removal, and then irradiated from the top side with a 300-W Xe lamp (≤420 nm). A flow of cooling water was used to maintain the reaction suspension at 288 K. The evolved gases were analyzed by gas chromatography (Agilent GC-7890A, MS-5A column, TCD, Ar carrier). Typically, the catalysts in several square centimeters are applied for photocatalysis. In photocatalytic HER, the evolved gases were analyzed after 5 h reactions. In overall water splitting reactions, the activities tested were normalized to 1 cm^2^.

## Supplementary information

Supplementary Information

## Data Availability

More relevant data sets generated during and/or analyzed during the current study are available from the first authors and corresponding authors on reasonable request. Source data are provided with this paper.

## Source data

Source Data
