# Supplementary material for: Bipolar charge collecting structure enables overall water splitting on ferroelectric photocatalysts
Source: Nat Commun. 2022 Jul 22;13:4245. doi: 10.1038/s41467-022-32002-y (PMC9307613; doi:10.1038/s41467-022-32002-y)
Supplement: Supplementary file 1 — Supplementary Information [file 41467_2022_32002_MOESM1_ESM.pdf]

Supplementary Information

Bipolar charge collecting structure enables overall water splitting on ferroelectric photocatalysts

*Yong Liu[1], Mingjian Zhang[2], Zhuan Wang[3], Jiandong He[1], Jie Zhang[1], Sheng Ye[1], Xiuli Wang[1], Dongfeng Li[1], Heng Yin[1], Qianhong Zhu[1], Huanwang Jing[4], Yuxiang Weng[3], Feng Pan[2], Ruotian Chen[1], Can Li[1,4], Fengtao Fan[1]**

[1]State Key Laboratory of Catalysis, Dalian National Laboratory for Clean Energy, iChEM, Dalian Institute of Chemical Physics, Chinese Academy of Sciences, Dalian 116023, China.

[2]School of Advanced Materials, Peking University, Shenzhen Graduate School, Shenzhen 518055, China.

[3]The Laboratory of Soft Matter Physics, Beijing National Laboratory for Condensed Matter Physics, Institute of Physics Chinese Academy of Science, Beijing 100190, China

[4]State Key Laboratory of Applied Organic Chemistry, Advanced Catalysis Center, College of Chemistry and Chemical Engineering, Lanzhou University, Lanzhou 730000, China

*Email: ftfan@dicp.ac.cn

Experiments

Solar energy conversion efficiency. The efficiency in this system is estimated via solar to hydrogen (STH) conversion efficiency,

$$STH = \frac{r_{H_2} \times \Delta G_{H_2O}}{P_{light} \times S} \tag{1}$$

r_{H_2} , ΔG_{H_2O}, P_{light}, and S represent the H_2 evolution rate, the reaction Gibbs energy of water splitting, the light energy flux and the sample area, respectively. Based on these, the STH conversion efficiency is less than 0.01 %.

Piezoelectric Force Microscopy (PFM). The PFM local piezoelectric hysteresis loops of the samples were measured using Piezoelectric force microscopy (PFM; Bruker) under ambient atmosphere in the Piezoelectric Force Microscopy (PFM) mode. The Pt/Ir-coated Si tip was used as a tip with a spring constant of 1–5 N/m at single PR drive frequency of about 370 KHz. Due to the single drive frequency and morphology roughness, the phase shift is found in the phase image.

Experiment of fs-Mid-IR TAS. A femtosecond amplifier laser system (Spitfire Ace, spectra Physics) which generated 35 fs laser pulses centered at 800 nm with a repetition rate of 1 kHz, was utilized as the light source. The output was split into three beams. The first beam was used to pump an optical parametric amplifier (TOPAS, spectra Physics) to generate excitation pulses. The second beam was utilized to generate midinfrared super-continuum. Briefly, after a second harmonic generation nonlinear crystal (BBO), the 800 nm fundamental light and the second harmonic at 400 nm was focused into air directly. With finely tuning the superposition of the two beams both in time and space, a mid-IR super continuum was generated by four wave mixing. The mid-IR probe light was detected by a liquid-nitrogencooled 64-element mercury-cadmium-telluride coupled spectrometer (iHR 320, HORIBA Jobin Yvon). The time delay between the pump beam and the probe beam was controlled by a motorized delay stage. During the fs-mid-IR TAS experiments, the pump beam intensity was set to be 300 uJ in an area of 0.07 mm^2. The fitting results of Supplementary Fig. 20 is shown below:

Supplementary Table 1 The fitting results of femtosecond time-resolved carrier decay kinetics

Sample	τ1	A1	τ2	A2	τ3	A3
BTO	114.88	0.000812	1743.63	0.00469	------------	------------
Au/BTO	133.02	0.000717	2129.81	0.00246	0.08	0.002164

It should be pointed out that the BTO single crystal is about 0.5 mm. The concentrated charges are condensed in less than 100 nm. Thus, hardly the charge transfer affects the TAS feature of BTO.

Calculation for steady state charge density. In order to obtain charge density around Au particle, we used Poisson equation

$$\nabla^2 V = \varphi/\varepsilon \quad (2)$$

to calculate the steady state charge density of Au/BTO in Figure 2a. The surface potential is based on the fitted CPD.

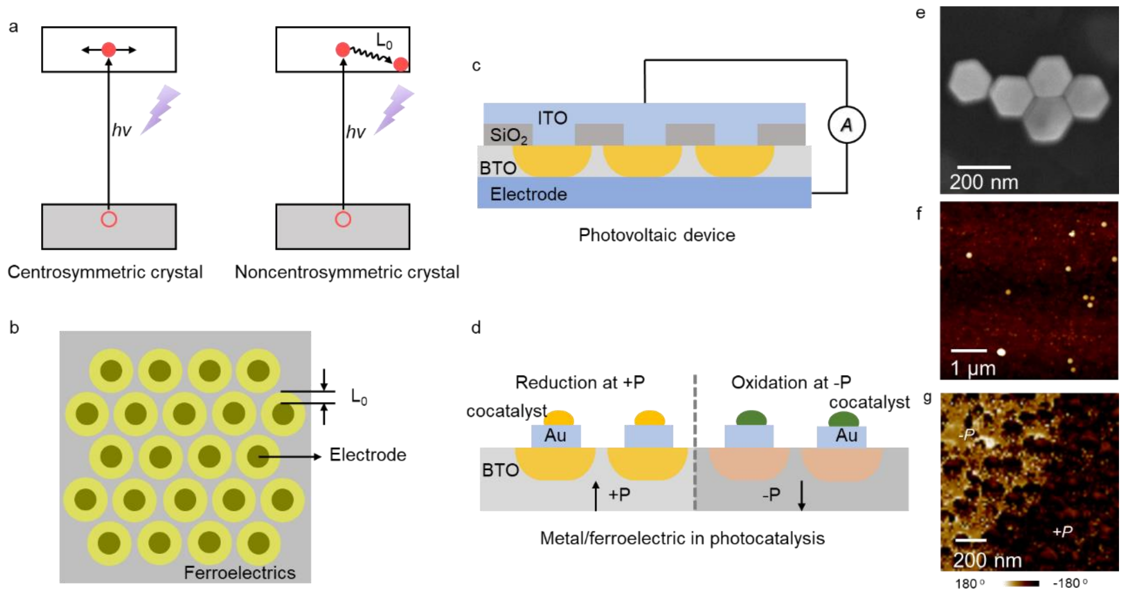

Supplementary Fig. 1 Schematic of utilization photogenerated charges in ferroelectrics. (a) Schematic illustrations of the photoexcitation processes in centrosymmetric crystal (left) and noncentrosymmetric crystal (right). (b) Schematic illustrations of hexagonal close-packed metallic electrode arrays with accurate distance on ferroelectrics substrate. (c) Schematic illustrations of ferroelectric photovoltaic device in Spanier's work. (d) Schematic illustrations of ferroelectric photocatalysis. (e) Scanning electron microscope (SEM) imaging of Au particles. Au particles are hexagonal nanoplate. Au particles about 200 nm in diameter and about 50 nm in thickness. Scale bar, 200 nm. (f) AFM topography of Au particles on BTO. Scale bar, 1000 nm. (g) Corresponding PFM phase of BTO in Fig. 1b. Due to the applied single frequency PFM, the phase show shift with topography, which is an inevitable phenomenon. Considering both PFM and KPFM results, we can confirm the antiparallel ferroelectric domains in the (001)-oriented BTO single crystal.

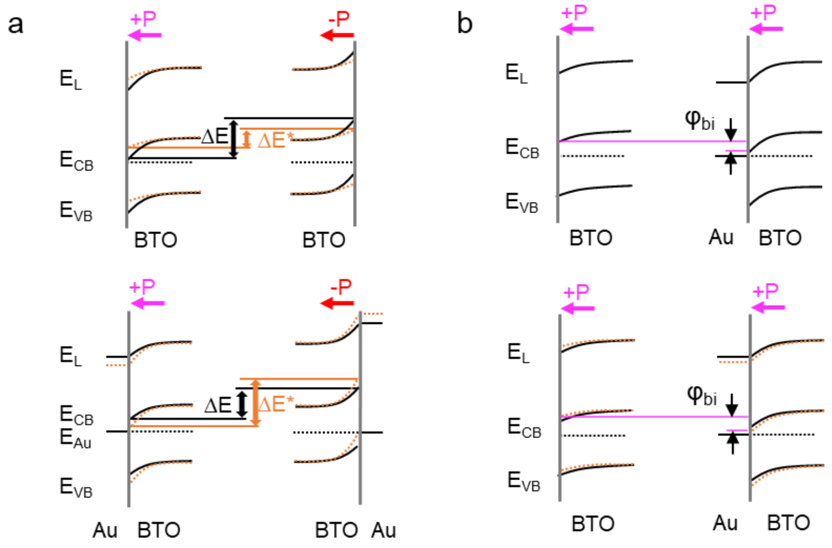

Supplementary Fig. 2 Schematic band bending of BTO and Au/BTO in the dark and under illumination. (a) Schematic measured LWF contrast between the two antiparallel ferroelectric domains of BTO (upper) and Au/BTO (lower) in dark and light. (b) Schematic measured φ_{bi} of Au/BTO interface in dark (upper) and light (lower). Solid black line, dark. Orange short dash, illumination. E_L, localized vacuum level. E_{CB}, conduction band. E_{VB}, valence band.

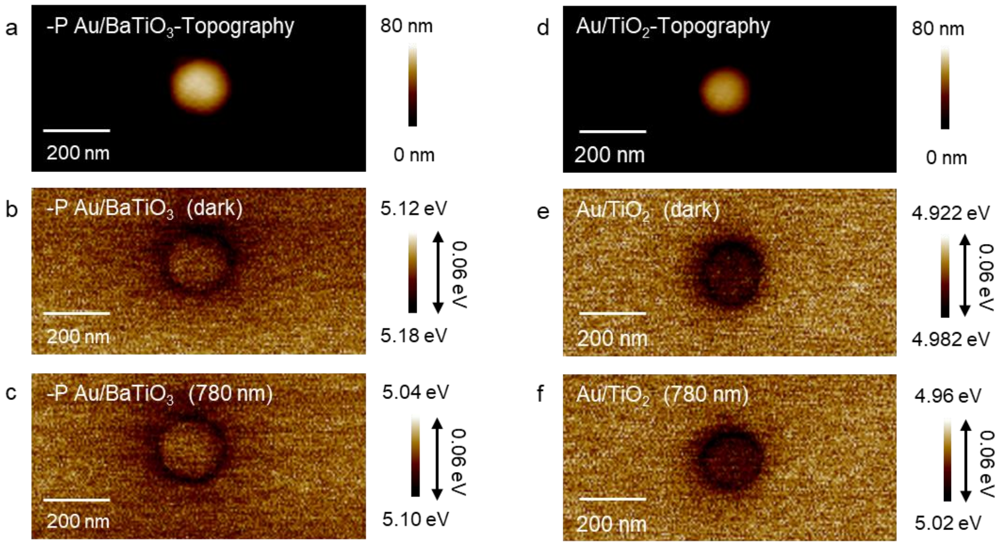

Supplementary Fig. 3 Charge separation of Au/$BaTiO_3$ and Au/TiO_2 under 780 nm light irradiation. (a) Topography of Au/BTO at -P. Scale bar, 200 nm. (b) LWF of Au/BTO in dark. Scale bar, 200 nm. (c) Surface potential of Au/BTO in 780 nm light. Scale bar, 200 nm. 780 nm is the surface plasmons resonance (SPR) peak of Au particle. The built-in voltage at the interface of Au/BTO remain the same. It indicates that no obvious charge transfer from Au particles to BTO substrate is observed when Au particles are irradiated. The LWF of BTO is decreased about 0.08 eV due to the sub-band irradiation of surface state or thermalization effect. (d) Topography of Au/TiO_2. Scale bar, 200 nm. (e) LWF of Au/TiO_2 in dark. Scale bar, 200 nm. (f) LWF of Au/TiO_2 in 780 nm light. Scale bar, 200 nm. 780 nm is the surface plasmons resonance (SPR) peak of Au particle. The built-in voltage at the interface of Au/TiO_2 remain the same. It indicates that no obvious charge transfer from Au particles to TiO_2 substrate is observed when Au particles are irradiated. The LWF of TiO_2 is increased about 0.04 eV due to the sub-band irradiation of surface state or thermalization effect.

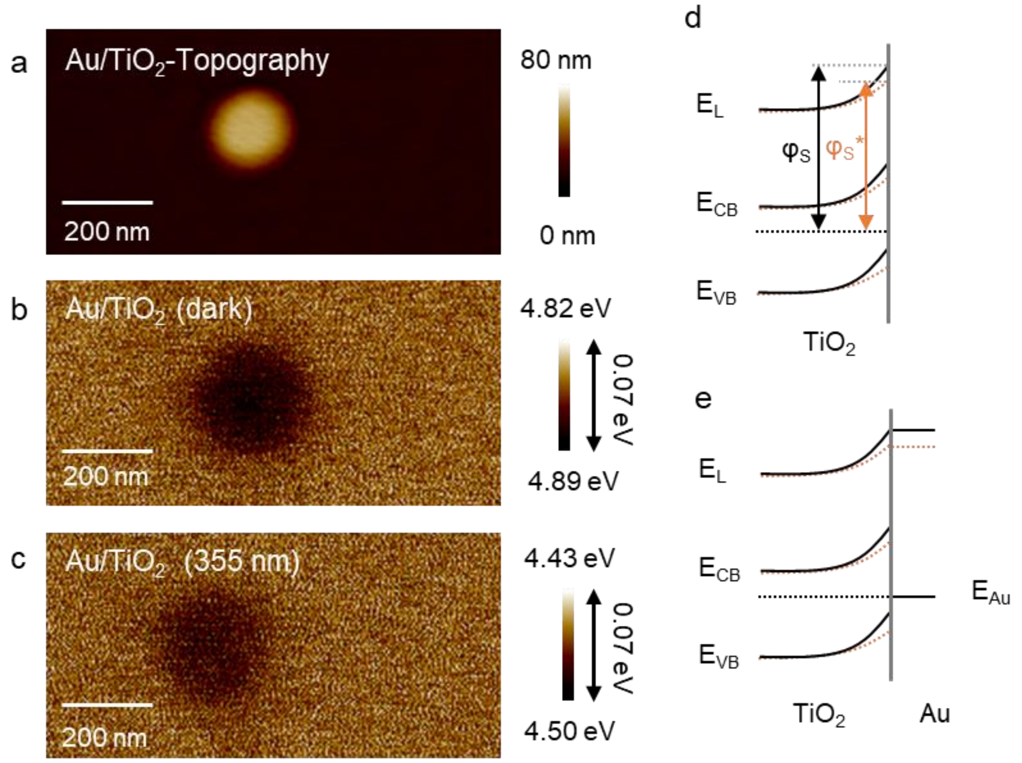

Supplementary Fig. 4 Charge separation of Au/TiO_2 under 355 nm light irradiation. (a) Topography of Au/TiO_2. Scale bar, 200 nm. (b) LWF of Au/TiO_2 in dark. Scale bar, 200 nm. (c) LWF of Au/TiO_2 in 355 nm light. Scale bar, 200 nm. Under 355 nm light, the TiO_2 is irradiated. The LWF at the interface of Au/TiO_2 almost remain the same. It indicates that no obvious charge transfer from Au particles to TiO_2 substrate is observed when Au particles are irradiated. The LWF of TiO_2 reduces about 0.4 eV due to the super-band irradiation of TiO_2 and the charge transfer to surface. (d) Schematic band bending of TiO_2 in the dark and under 355 nm light irradiation. Black solid line, in dark. Orange dashed line, under 355 nm light. irradiation. (e) Schematic band bending of Au/TiO_2 in the dark and under 355 nm light irradiation. Black solid line, in dark. Orange dashed line, under 355 nm light. irradiation.

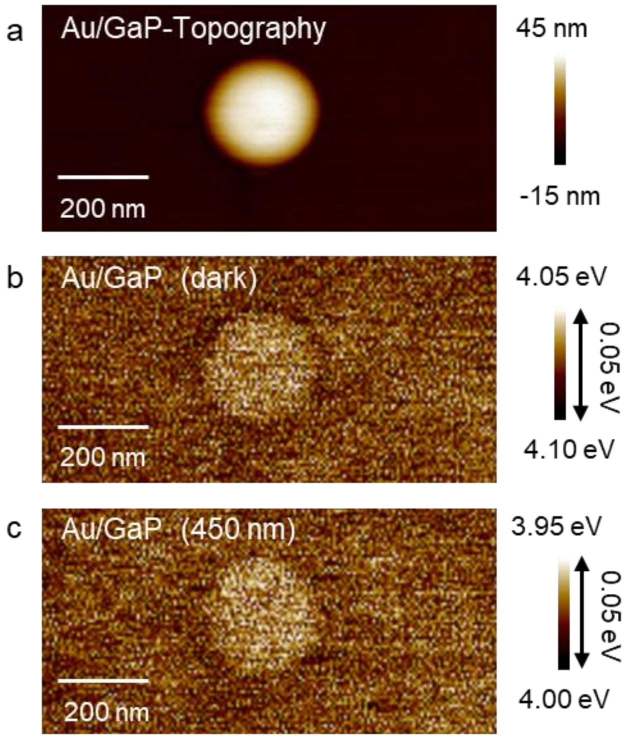

Supplementary Fig. 5 Charge separation of piezoelectric Au/GaP under 450 nm light irradiation. (a) Topography of Au/GaP. Scale bar, 200 nm. (b) LWF of Au/ GaP in dark. Scale bar, 200 nm. (c) LWF of Au/ GaP in 450 nm light. Scale bar, 200 nm. Under 450 nm light, the GaP is irradiated. The built-in voltage at the interface of Au/ GaP remains the same. It indicates that no obvious charge transfer from Au particles to GaP substrate is observed when Au particles are irradiated. The LWF of TiO_2 is increased about 0.1 eV due to the super-band irradiation of GaP.

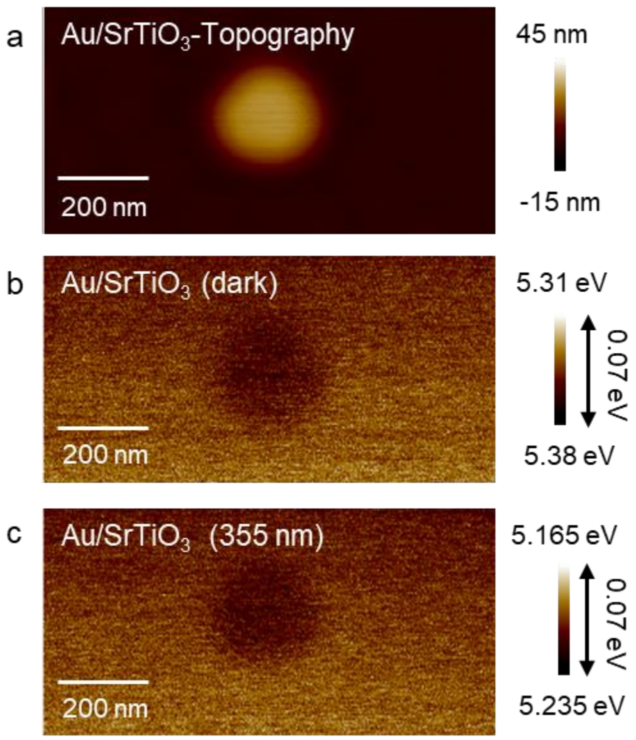

Supplementary Fig. 6 Charge separation of perovskite semiconductor Au/$SrTiO_3$ under 355 nm light irradiation. (a) Topography of Au/$SrTiO_3$. Scale bar, 200 nm. (b) LWF of Au/ $SrTiO_3$in dark. Scale bar, 200 nm. (c) LWF of Au/ GaP in 450 nm light. Scale bar, 200 nm. Under 450 nm light, the GaP is irradiated. The built-in voltage at the interface of Au/ GaP remains the same. It indicates that no obvious charge transfer from Au particles to GaP substrate is observed when Au particles are irradiated. The LWF of $SrTiO_3$ is increased about 0.145 eV due to the super-band irradiation of $SrTiO_3$.

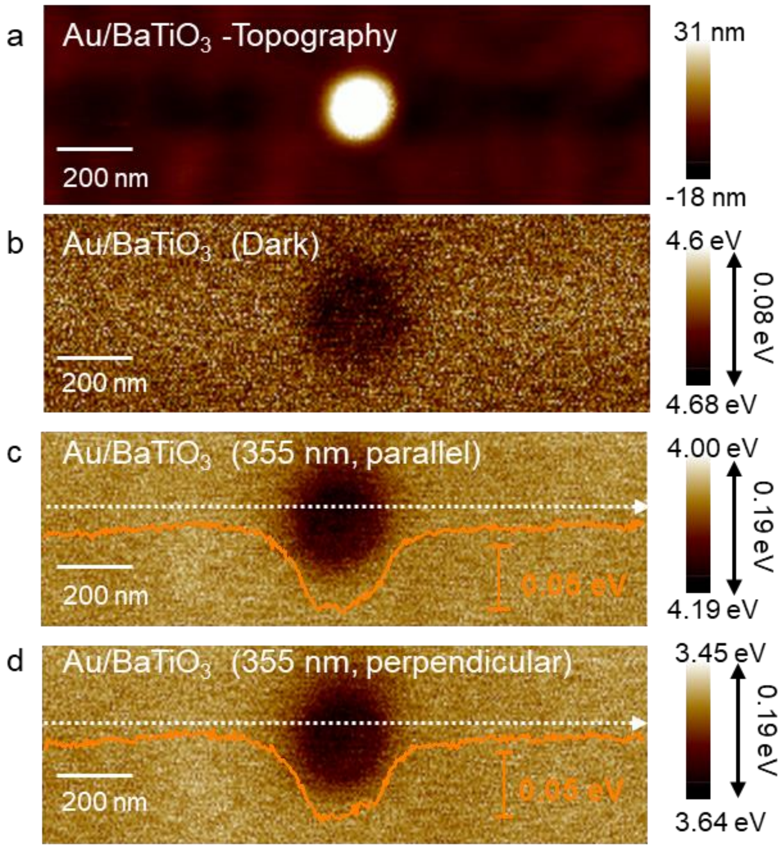

Supplementary Fig. 7 Bulk photovoltage effect at Au/$BaTiO_3$. (a) Topography of Au/BTO at -P. Scale bar, 200 nm. (b) LWF of Au/BTO in dark. Scale bar, 200 nm. (c) LWF of Au/BTO in 355 nm light. The polarization direction of 355 nm light is parallel to that of BTO. Scale bar, 200 nm. (d) LWF of Au/BTO in 355 nm light. The polarization direction of 355 nm light is perpendicular to that of BTO. Scale bar, 200 nm. Under 355 nm light, the BTO is irradiated. The polarization direction of linearly polarization light is rotated via a half wave plate.

When the direction of light polarization is changed, the LWF of overall Au/BTO is increased about 0.55 eV due to the bulk photovoltage effect (BPVE) of BTO under linearly polarized light. The BTO single crystal is in the form of polydomain. As a result, the BPVE cause an overall LWF shift. However, the built-in voltage at the interface of Au/BTO remains the same. It indicates that no obvious charge transfer between Au particles to BTO substrate is observed. In addition, plasmonic charge diffusion preferentially aligns with the electric field direction of the polarized light[6, 7]. This phenomenon also indicates that the anomalous concentration of photogenerated charges

is independent of SPR effect. In conclusion, the discussion and analysis above not only exclude the effect of SPR effect but also confirm the key role of ferroelectricity in the anomalous concentration of photogenerated charges at the interface of ferroelectric Au/$BaTiO_3$.

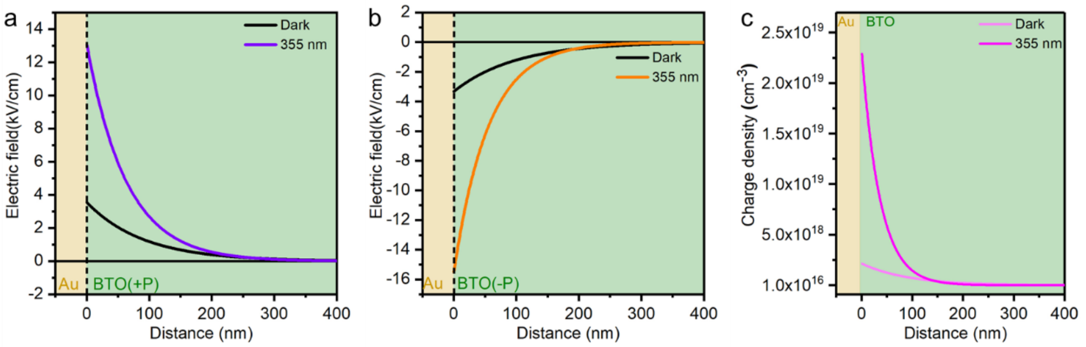

Supplementary Fig. 8 Calculated electric field intensity and steady state charge density. (a) Corresponding electric field intensity calculated from the differential of CPD with the distance away from Au at +P in IFG. 2a and 2b. (b) Corresponding electric field intensity calculated from the differential of CPD with the distance away from Au at -P in IFG. 2c and 2d. (c) Estimate of steady state charge density in dark and light in Supplementary Fig. 2a and 2b. Under illumination, the steady state charge density increases from 10^{16} cm^{-3} to 10^{19} cm^{-3}.

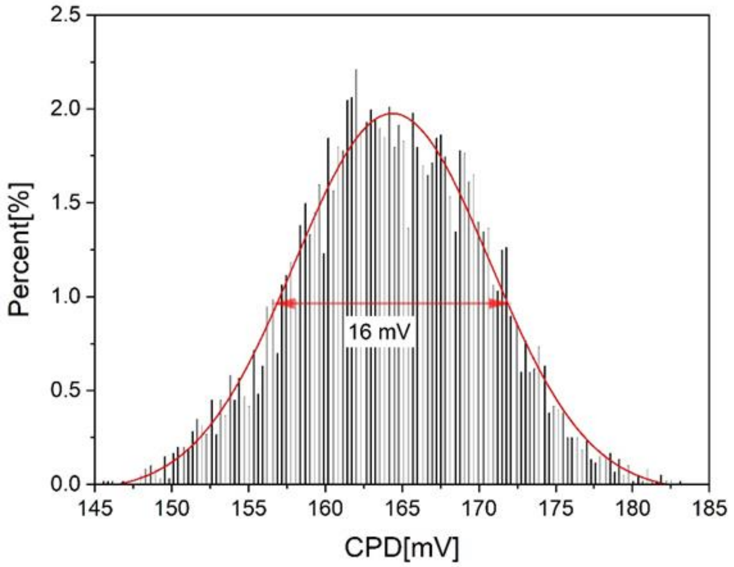

Supplementary Fig. 9 Statistical CPD distribution measured from freshly peeled off HOPG and fitted Gaussian distribution. The full width at half maximum value of the images' Gaussian distribution is about 16 mV. The CPD value is around 164±16 mV.

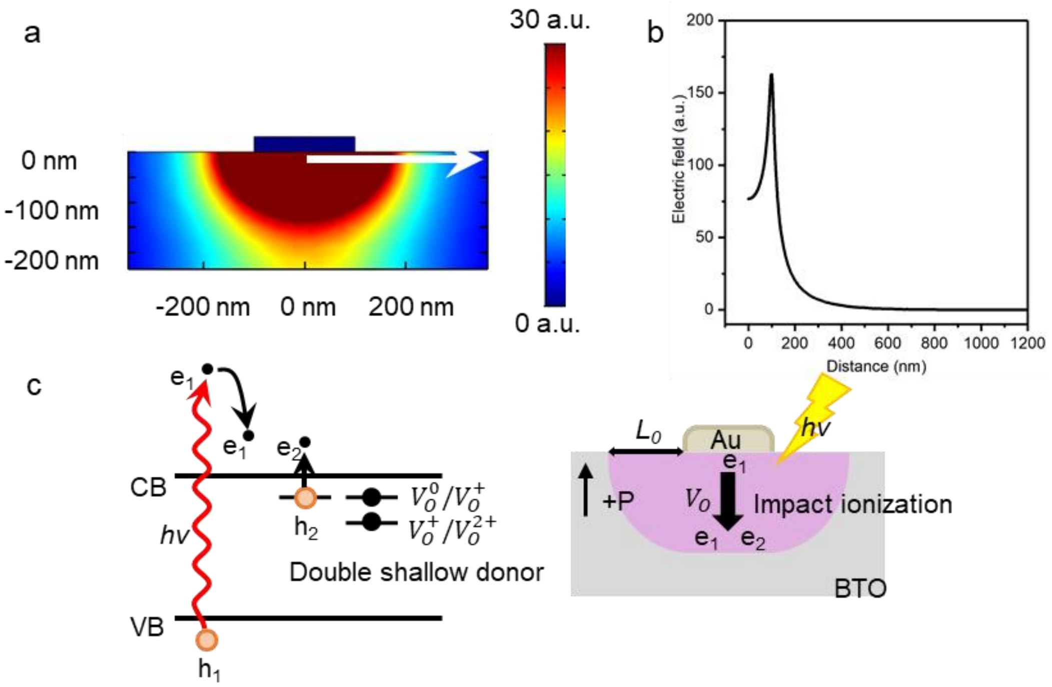

Supplementary Fig. 10 Schematic of intense electric field around Au. (a) Simulation of enhanced electric field around Au at Au/BTO interface. The field expands radially in all directions in BTO and thus coming into being a slightly horizontally stretched hemisphere. Quantificationally, the enhanced field extend horizontally about 150 nm and away from the margin of Au particle, which is more extensive than measured L_0. This electric field contribute for impact charge ionization. But the width of the SCR under illumination is determined by the thermalization length L_0. (b) Electric field intensity values extracted across the white line in Supplementary Fig. 10a. (c) Schematic mechanism for charge separation at Au/BTO interface. The electron-occupied states are about 0.1-0.5 eV below the conduction band minimum (CBM). Coupled with ballistic mechanism of BTO, impact ionization of oxygen vacancies is occurred within the enhanced electric field. As a result, impact ionization occurs easily with the help the enhanced electric field around Au[8, 9]. Under illumination, a photon produces the first pair of electron e_1 and hole h_1 from the oxygen vacancy. e_1 with high-mobility relaxes and produces a second pair of electron e_2 and hole h_2. As a result, photogenerated charges are concentrated at the SCRs beneath BTO within a hemisphere of radius L_0 about 50 nm around Au.

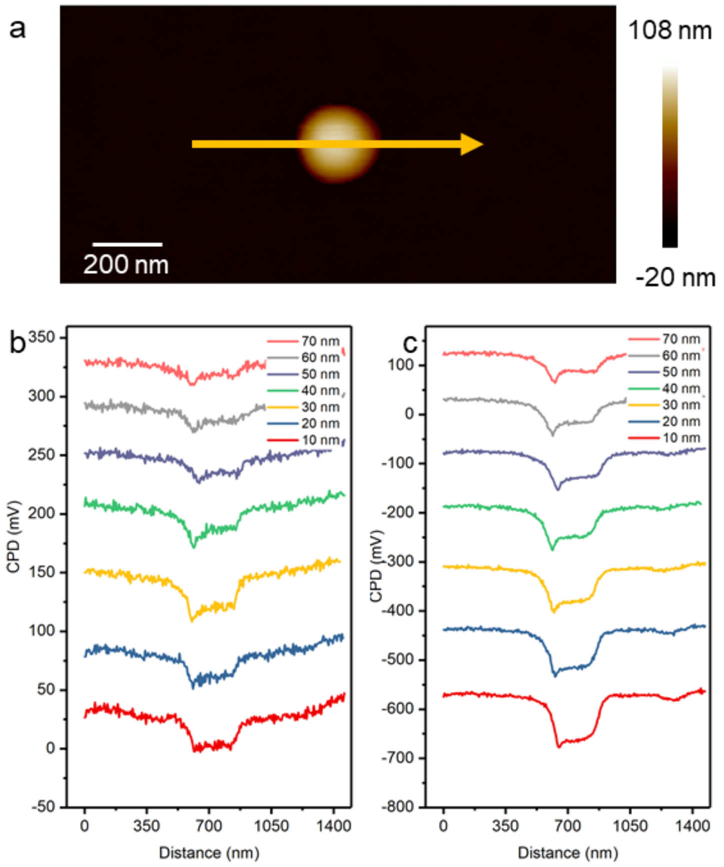

Supplementary Fig. 11 Surface potential waterfall at different lift height. (a) Topography and surface potential. (b, c) Waterfall surface potential value extracted across the line of different lift height in dark (b) and light (c), respectively. The lower the lift height is, the worse the signal to noise ratio is. The surface potential at 10 nm lift height is the same with that at 20 nm. Considering both signal to noise ratio and distortion, we choose 20 nm lift height.

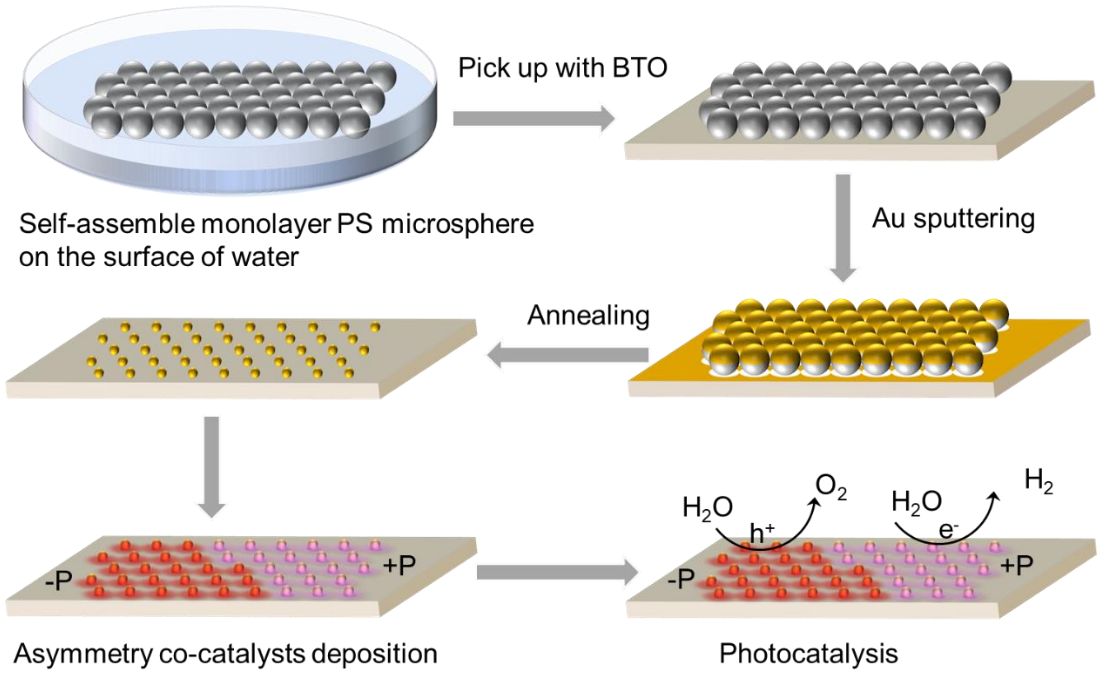

Supplementary Fig. 12 Schematic of preparing Au array/BTO photocatalysts. Firstly, self-assemble monolayer PS microsphere is dispersed on the surface of water. And then, the monolayer PS microsphere is picked up with hydrophilic BTO single crystal substrate. Next, Au film with proper thickness is deposited. After annealing in 1000 ºC, the Au film is transformed into hcp Au particles. Subsequently, the co-catalysts are spatially deposited under illumination. The prepared ferroelectric photocatalyst can be applied for overall water splitting. For different Au arrays density, different size PS microspheres are used. And different Au film thicknesses are sputtering to ensure the Au particles of different samples of similar size.

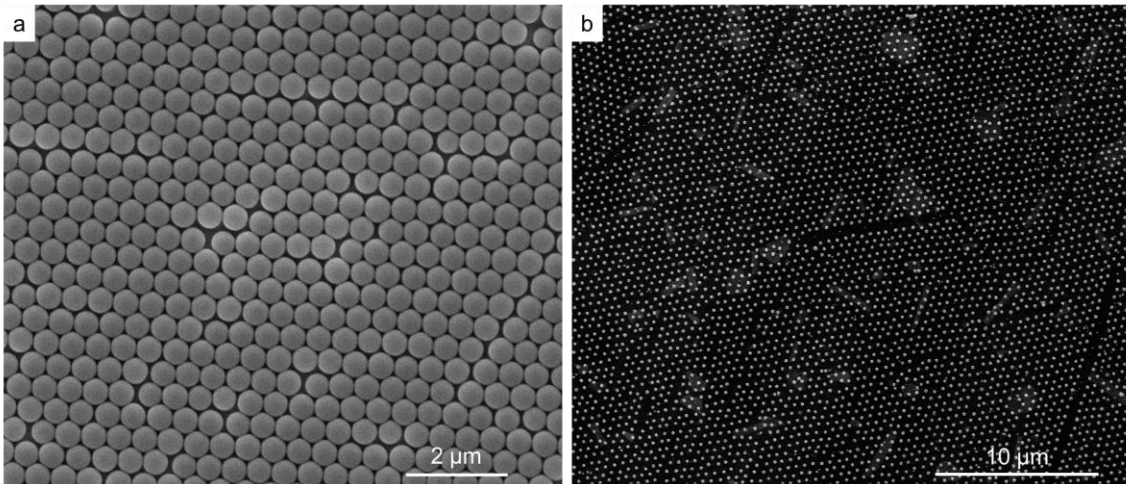

Supplementary Fig. 13 SEM images of PS microsphere and Au arrays on BTO. (a) Monolayer hcp PS microsphere on BTO. (b) hcp Au array on BTO.

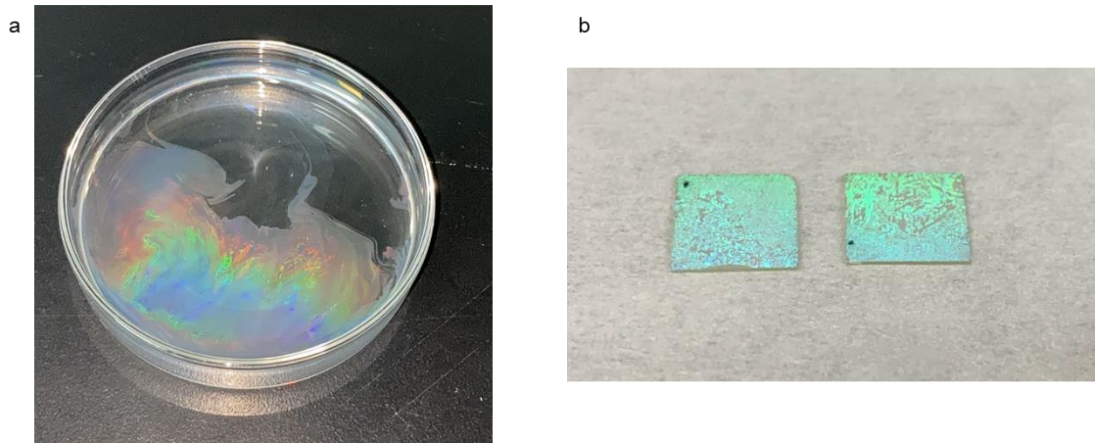

Supplementary Fig. 14 Pictures of self-assemble monolayer PS microsphere. (a) PS microsphere on water surface. (b) PS microsphere on BTO single crystal. The Bragg diffraction indicate the periodic nano structure in large scale.

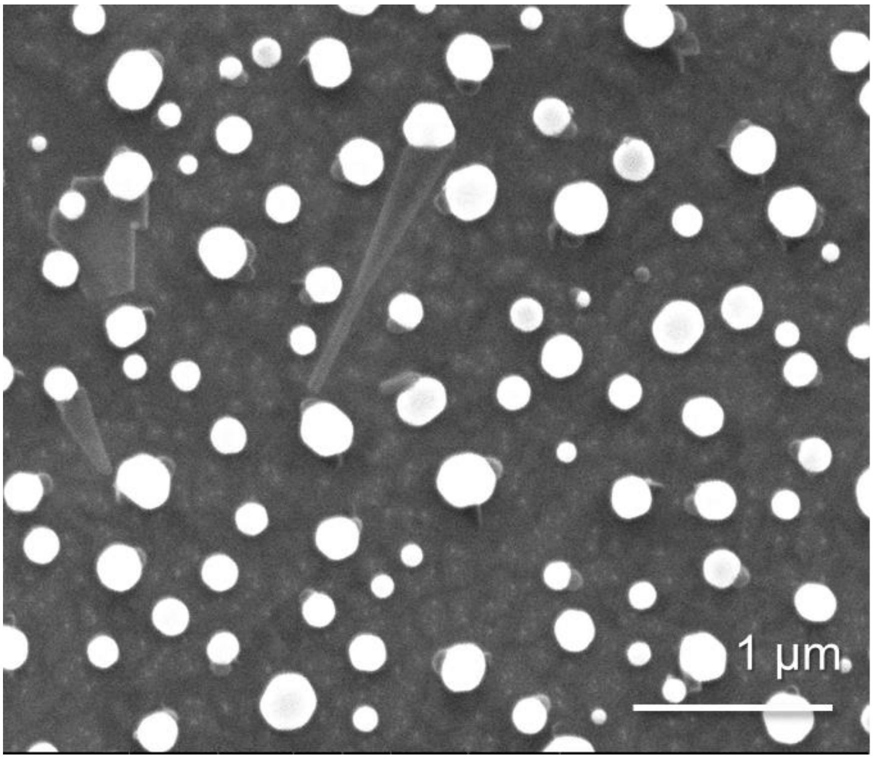

Supplementary Fig. 15 Au film on BTO after annealing without PS microsphere. The diameter of Au particles ranges from 10 nm to 500 nm with random distance.

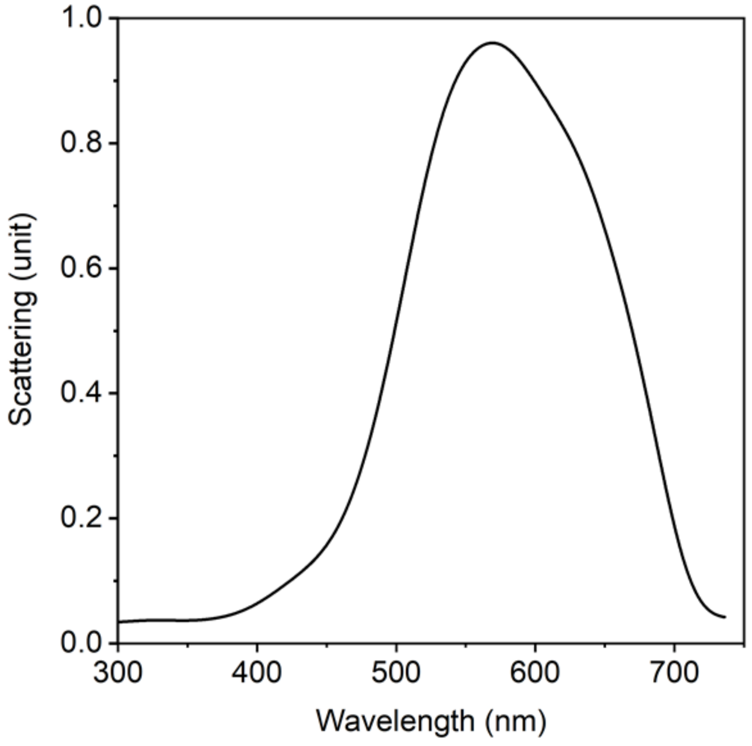

Supplementary Fig. 16 Dark-field scattering spectrum of single Au array/BTO. The spectrum is taken under unpolarized white light illumination in optical microscopy.

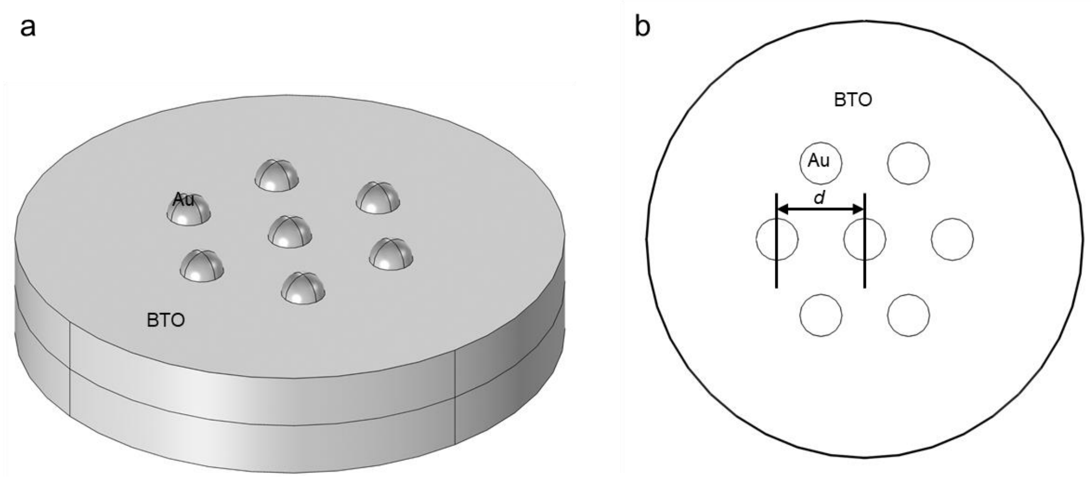

Supplementary Fig. 17 Schematic of fabricated model of Au array on BTO in simulation. (a) 3D view of Au particles on BTO. (b) Top view. The BTO cylinder is 500 nm in height and 1250 nm in radius. The Au particles is in radius of 100 nm. The size of BTO is over 10 time larger than that of Au particles. The distance between the two particles is *d*. The simulations are performed for *d*=500 nm and *d*=300 nm, respectively. When *d*=300 nm, the distance between the margin of the adjacent Au particles is 100 nm, which is approximate twice of the measured L_0.

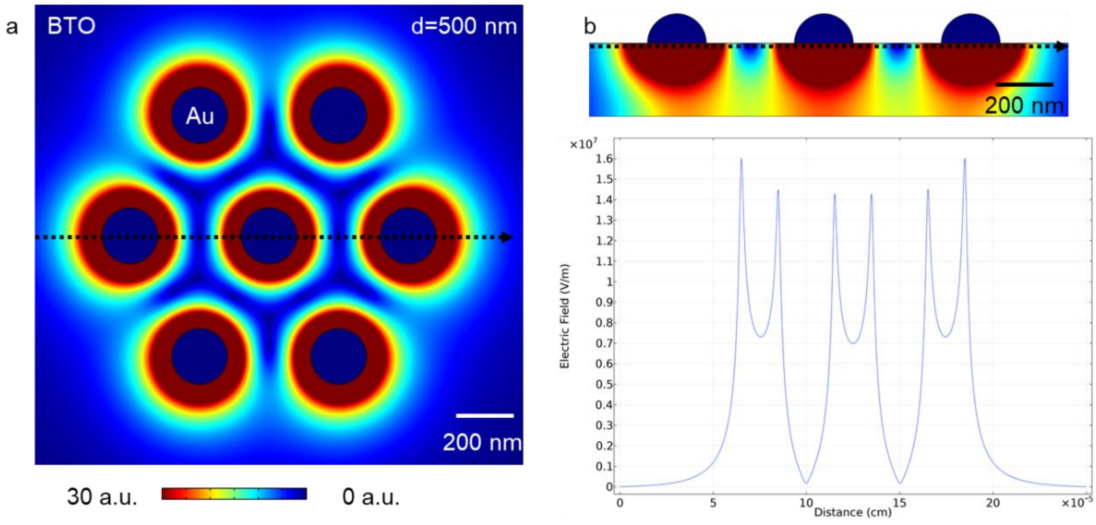

Supplementary Fig. 18 Simulated electric field intensity when *d* is 500 nm. (a) Top view. (b) Side view (up) and electric field intensity cross the line. The field around the outside Au particle is asymmetrically deformed as a result of the electrostatic repulsion. The enhanced electric field around Au particles is obviously contracted horizontally, comparing with a single Au particle in Supplementary Fig. 10.

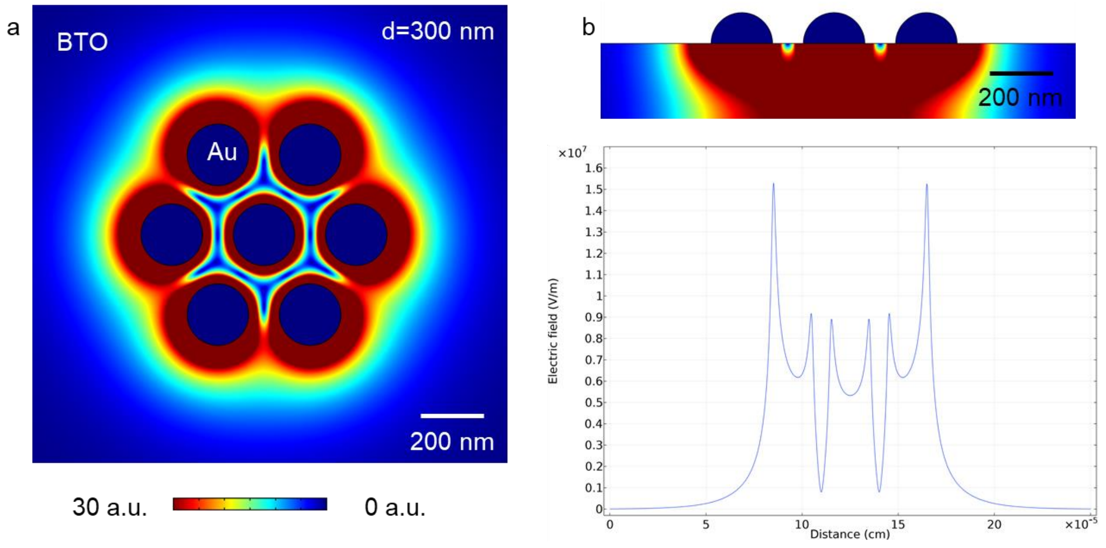

Supplementary Fig. 19 Simulated electric field intensity when *d* is 300 nm. (a) Top view. (b) Side view (up) and electric field intensity cross the line. Compared with *d*=500 nm in Supplementary Fig. 18, the electric field around the Au particles extends much narrower and weaker when *d* is 300 nm. The radially-expanded electric field is significantly deformed.

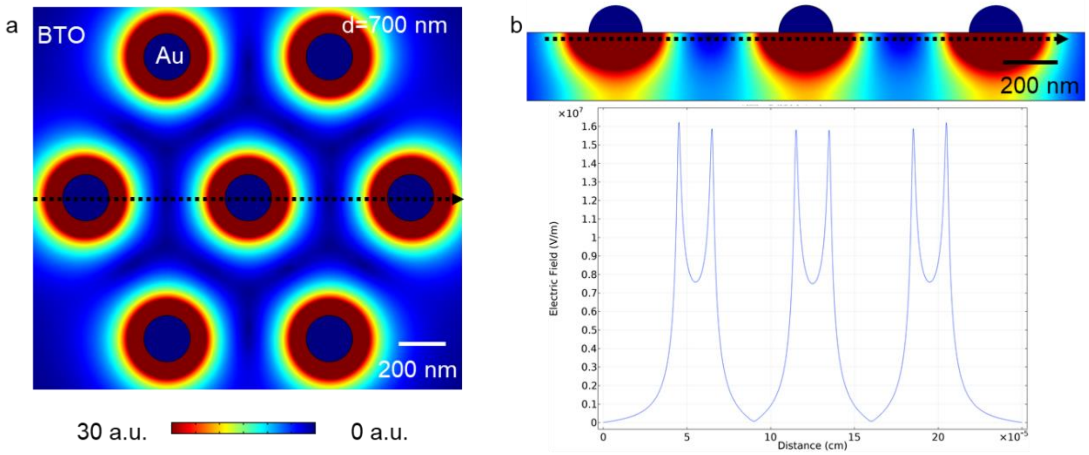

Supplementary Fig. 20 Simulated electric field intensity when *d* is 700 nm. (a) Top view. (b) Side view (up) and electric field intensity cross the line. Compared with *d*=500 nm in Supplementary Fig. 18, the electric field around the Au particles extends almost the same when *d* is 700 nm.

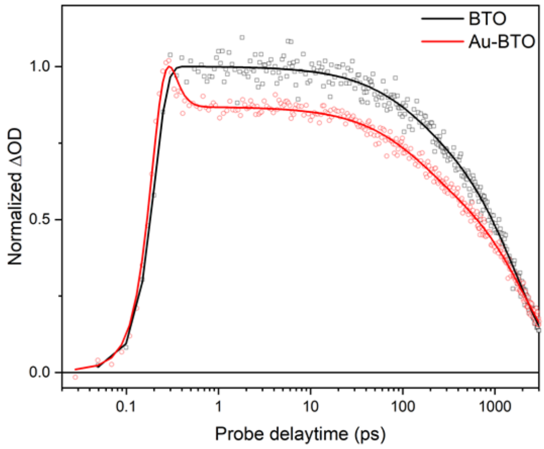

Supplementary Fig. 21 fs-mid-IR transient absorption spectroscopy (fs-mid-IR TAS) of BTO and Au array/BTO, pumped at 355 nm, probed at 5000 nm (Details in Supporting Information). The signal in mid-IR is mainly originated from photogenerated free charges (or nonthermalized charges) in semiconductors instead of metals[10, 11]. Comparing the fs-mid-IR TAS feature of BTO and Au/BTO, an ultrafast decay of 0.08 ps is found on Au/BTO. Au particles at this size show no fs-TAS features and are transparent at 5000 nm probe[11, 12]. In this spectral region, spectral overlap between the BTO and Au TAs features can be avoided. Thus, this ultrafast decay is attributed to BTO, validating the nonthermalized electrons transferring from BTO to Au[11]. Together with the photodeposition experiments in Supplementary Fig. 3, we confirm the electrons transfer from BTO to Au at +P.

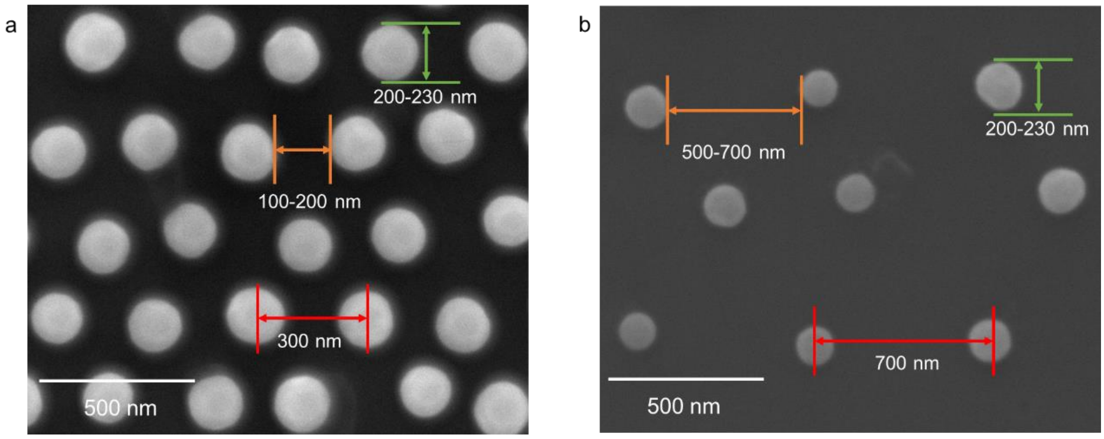

Supplementary Fig. 22 SEM images of BTO/Au. (a) BTO/Au300 (b) BTO/Au700.

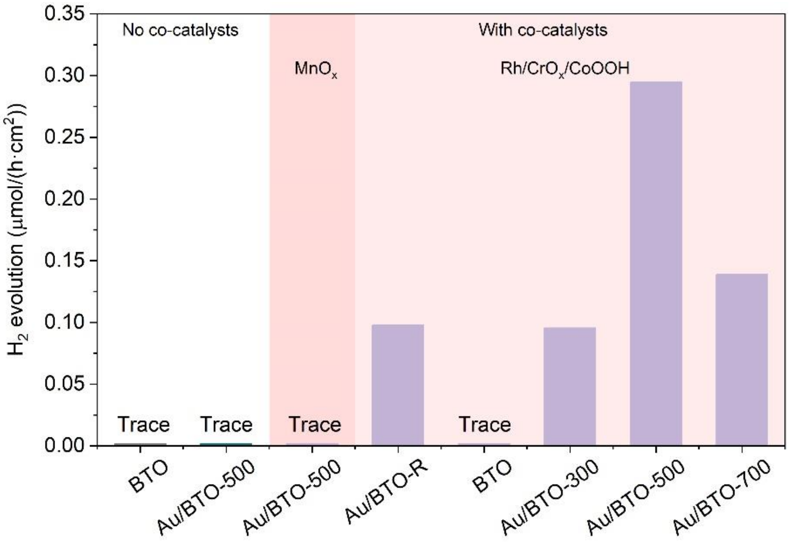

Supplementary Fig. 23 Hydrogen evolution of overall water splitting (OWS) in pure water.

Without constructed Au/BTO junction and proper cocatalysts (MnOx cocatalyst), the OWS will not occurred in pure water. With 500 nm spacing, the Au/BTO500 show the highest OWS activity after photodeposited Rh/CrOx/CoOOH cocatalysts. With 300 nm spacing, the Au/BTO500 show dramatically reduced activity due to the electrostatic repulsion between the neighbour Au particles. With 700 nm spacing, the water splitting performance of Au/BTO700 is lower than that of Au/BTO500 due to the lower density of Au particles in Au/BTO700. With random spacing, Au/BTOR also show obvious decreased activity due to random spacing. These control experiments demonstrated the importance of enhanced charge separation beyond the thermalization length and the enhanced electric field at Au/BTO interface.

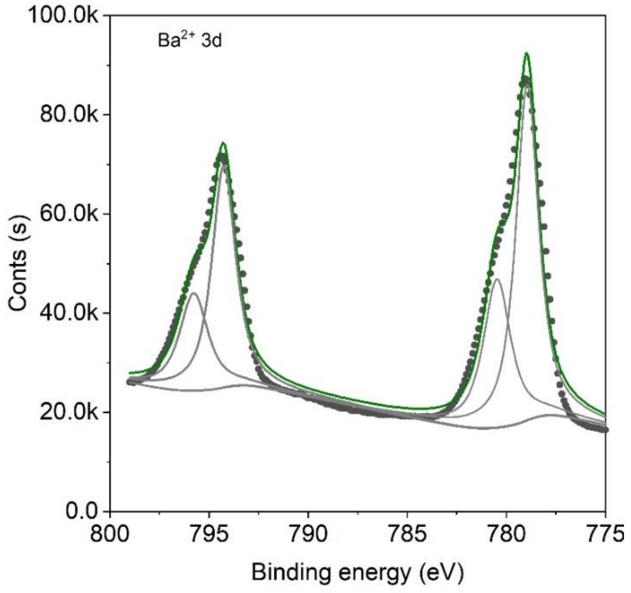

Supplementary Fig. 24 XPS of Ba^{2+} 3d peaks. There are obvious two states of Ba, the one with higher binding energy (780.4 eV) and the one with lower binding energy (778.9 eV), corresponding to the polydomain structure of $BaTiO_3$[13].

Discussion about oxygen vacancies in BTO: (Supplementary Fig. 25-29)

Oxygen vacancies in $BaTiO_3$ can be altered via annealing in air. And oxygen vacancy is also the mean reason why the BTO is an n-type semiconductor[14, 15]. A control sample with 3h annealing, less oxygen vacancies, is prepared, which is conformed via XPS (Supplementary Fig. 25).

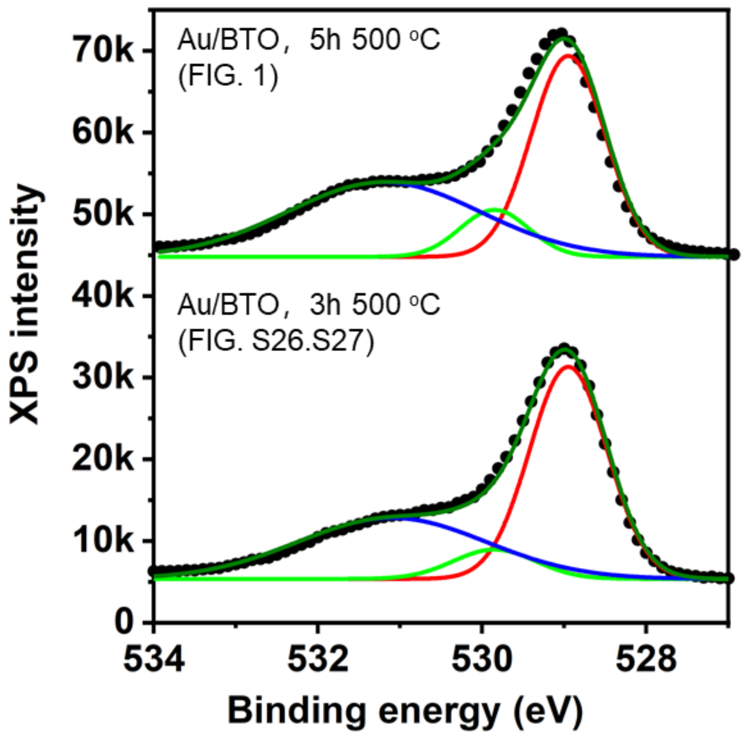

Supplementary Fig. 25 XPS of O 1s of Au/BTO (in Supplementary Fig. 26 and 27). Oxygen vacancy, green line. Absorption oxygen, blue line. O^{2-}, red line. The concentration of oxygen vacancy in Au/BTO (500 °C, 5h) was obviously higher than that of Au/BTO (500 °C, 3h).

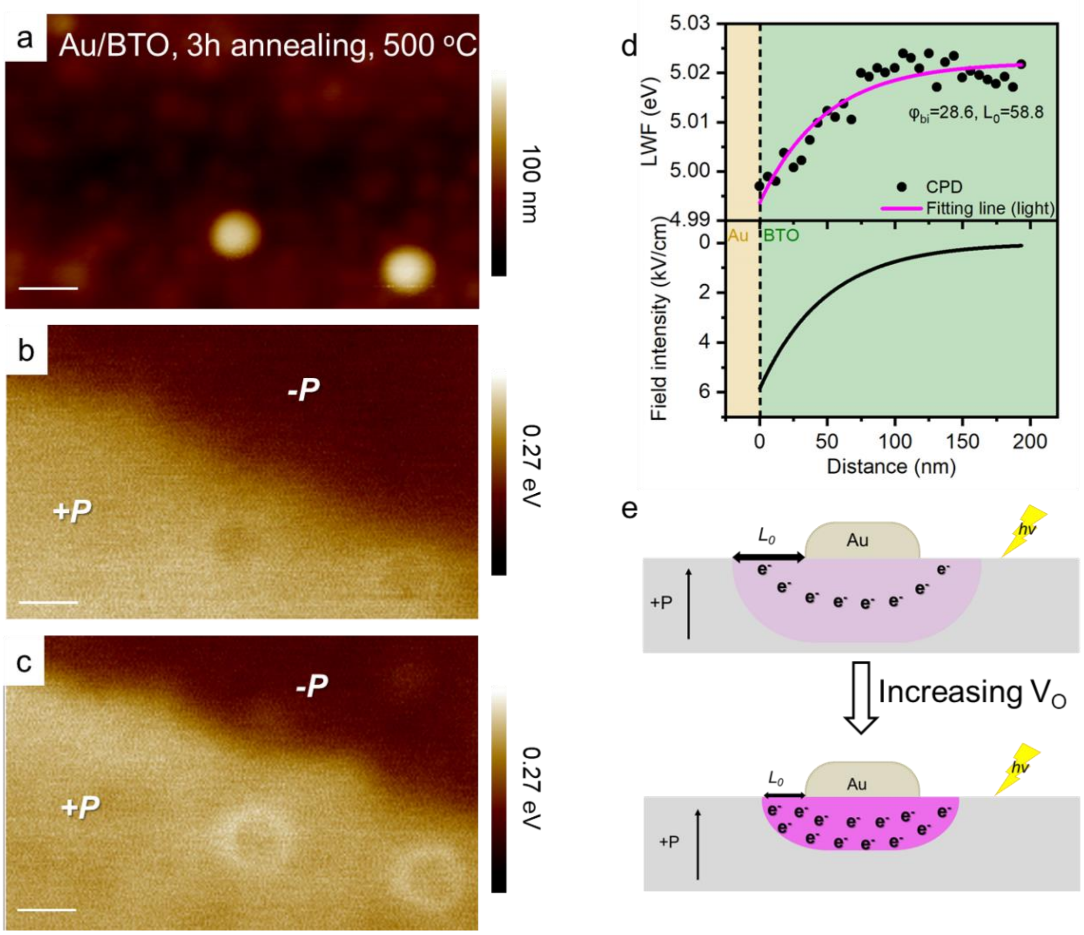

Supplementary Fig. 26 Topography and surface potential of Au/BTO (500 °C, 3h annealing) at +P. a) Topography of Au/BTO at +P. Scale bar, 200 nm. b) LWF of Au/BTO at +P in dark. No additional built-in voltage is formed at Au/BTO interface. Scale bar, 200 nm. c) LWF of Au/BTO at +P under 355 nm laser illumination. An additional built-in voltage is formed. Scale bar, 200 nm. d) LWF and electric field intensity at Au/BTO interface of +P in light. Au/BTO interface is the null point. The SCR at Au/BTO interface increases and the built-in voltage deceases, compared to Au/BTO in Supplementary Fig. 1 and Fig. 2e) Schematic of photogenerated electrons concentrating around Au particle at +P of Au/BTO and Au/BTO. When the concentration of oxygen vacancy increases, photogenerated electrons are concentrated in a narrower SCR and the thermalization length decreased.

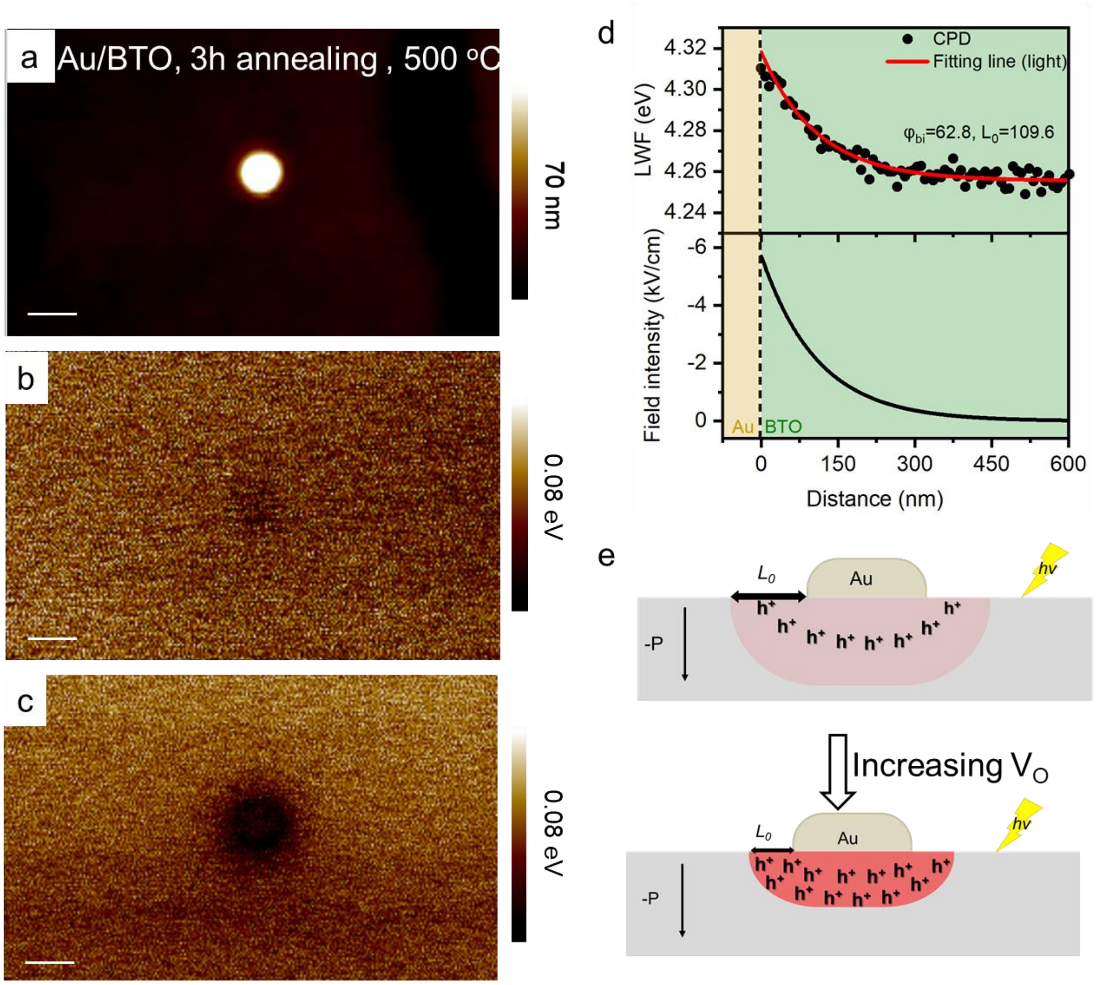

Supplementary Fig. 27 Topography and surface potential of Au/BTO (500 ºC, 3h annealing) at -P. a) Topography of Au/BTO at -P. Scale bar, 200 nm. b) LWF of Au/BTO at -P in dark. No additional built-in voltage is formed at Au/BTO interface. Scale bar, 200 nm. c) LWF of Au/BTO at -P under 355 nm laser illumination. An additional built-in voltage is formed. Scale bar, 200 nm. d) LWF and electric field intensity at Au/BTO interface of +P in light. Au/BTO interface is the null point. The SCR at Au/BTO interface increases and the built-in voltage deceases, compared to Au/BTO in Supplementary Fig. 1 and Fig. 2e) Schematic of photogenerated electrons concentrating around Au particle at -P of Au/BTO and Au/BTO. When the concentration of oxygen vacancy increases, photogenerated electrons are concentrated in a narrower SCR and the thermalization length decreased.

The SCR at Au/BTO interface under illumination is depending on the concentration of oxygen vacancy: denser photogenerated charges are concentrated

around Au in a narrower thermalization length when the concentration of oxygen vacancy increased. Meanwhile, a more intensive electric field is there. When the oxygen vacancy concentration decrease, the SCRs of accumulation layer under illumination increased from 52.3 nm (Fig. 2) to 58.8 nm (Supplementary Fig. 26), the depleting layer under illumination increased from 55.1 nm (Fig. 2) to 109.6 nm (Supplementary Fig. 27). Oxygen vacancy play a vital role in the anomalous concentration of photogenerated charges.

Supplementary Table 2 LWF fitting results of Au/BTO (5h, 500 °C) and Au/BTO (3h, 500 °C)

	$V_{bi\text{-}light}$ (mV)		$L_{0\text{-}light}$ (nm)	
	+P	-P	+P	-P
Au/BTO (5h, 500 °C)	78.5 (Fig. 2)	85.6 (Fig. 2)	52.3 (Fig. 2)	55.1 (Fig. 2)
Au/BTO (3h, 500 °C)	28.6 (Supplementary Fig. 26)	62.8 (Supplementary Fig. 27)	58.8 (Supplementary Fig. 26)	109.6 (Supplementary Fig. 27)

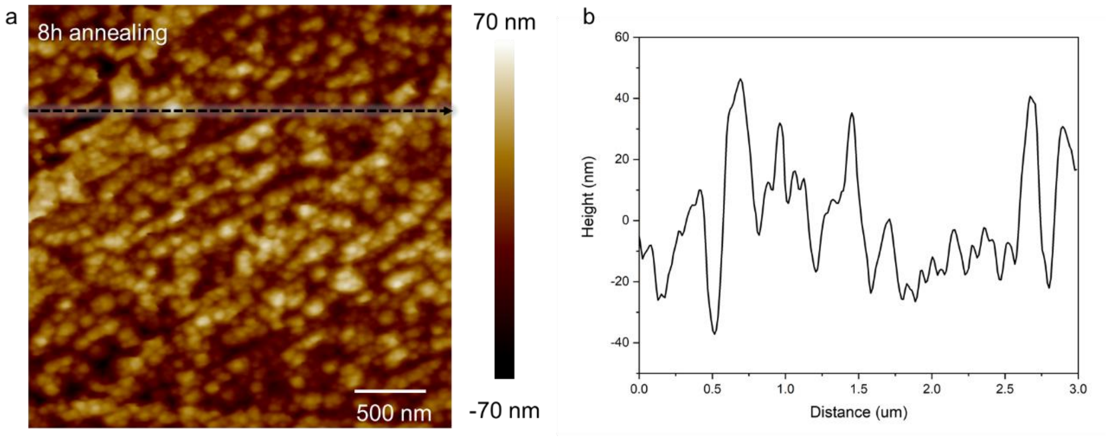

Supplementary Fig. 28 Topography of Au/BTO after 8 h annealing at 500 ºC (a) and cross line of height(b). When the annealing time is further prolonged to 8 h and the oxygen vacancies continued to increase, the surface of Au/BTO is deformed. The roughness of BTO is more the 80 nm, which is larger than the height of Au particles (50-80 nm). It is hard to identify the Au particle from topography and surface potential. The BTO surface is reconstructed.

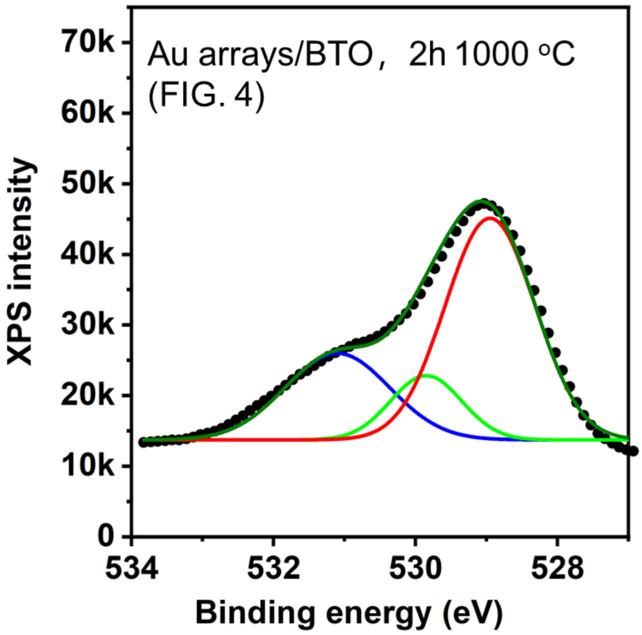

Supplementary Fig. 29 XPS of O 1s, Au array/BTO (in Supplementary Fig. 4) after 2h annealing at 1000 °C, whose oxygen vacancies are almost the same as Au/BTO (5h, 500 °C). Oxygen vacancy, green line. Absorption oxygen, blue line. O^{2-}, red line.

We demonstrate the oxygen vacancies in Au/BTO (500 °C 3h, Fig. 1) and Au arrays/BTO (1000 °C 2h, Fig. 4) are similar, based on oxygen vacancies amount and well-constructed Au/BTO junction in XPS. The thermalization length should be about 50 nm. Constructed Au arrays on BTO based on 50 nm thermalization length also show the highest water splitting.

In our present prepared methods, it is hard to control the oxygen vacancies more precise. More elaborate investigations may be done pulse laser depositing (PLD) or metal–organic decomposition (MOD), during which the oxygen vacancies can be control via oxygen pressure [16, 17].

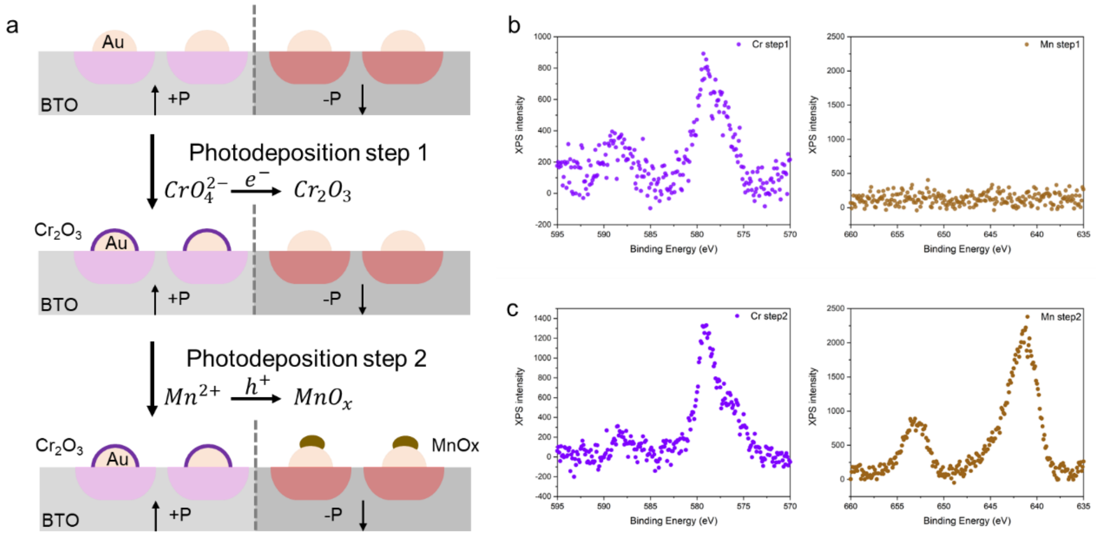

Supplementary Fig. 30 XPS of Cr_2O_3 and MnO_x after successively photodeposition of Cr_2O_3 and MnO_x. a) Schematic diagram of successively photodeposition of Cr_2O_3 and MnO_x. b) After photodeposition Cr_2O_3. c) After photodeposition MnO_x. Cr_2O_3 and MnO_x are successively photodeposited. Cr_2O_3 and MnOx are selectively deposited at different site, +P and -P, respectively.

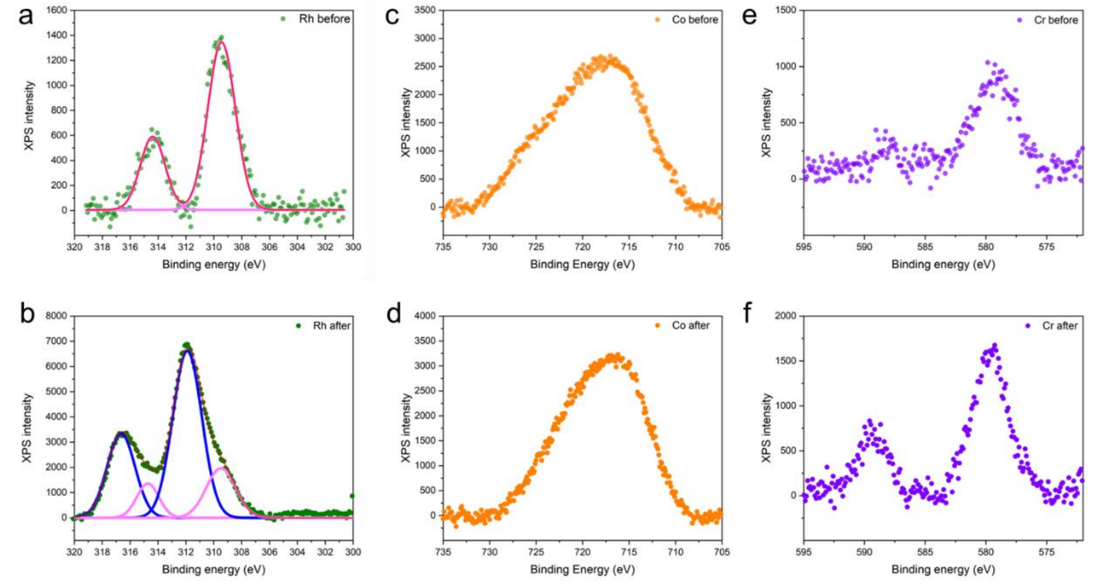

Supplementary Fig. 31 XPS of photodeposited Rh/Cr_2O_3/CoOOH co-catalysts before and after OWS. a) Rh 3d peaks of fresh cocatalysts before OWS. b) Rh 3d peaks of cocatalysts after OWS. c) Co LMM Auger peak of fresh cocatalysts before OWS. d) Co LMM Auger peak of cocatalysts after OWS. e) Cr 2p peaks of fresh cocatalysts before OWS. f) Cr 2p peaks of cocatalysts after OWS.

In Supplementary Fig. 31 a and b, Rh $3d_{5/2}$ XPS peaks at 309.4 eV indicates the Rh element in the fresh co-catalysts is in the form of Rh^0 metal. After OWS reaction, another peak at 311.9 eV (higher binding energy) emerges, corresponding to the Rh^{3+}.[18-20]

The binding energy of Ba 3d and Co 2p are overlapped. And thus, the Co LMM Auger peak are displayed in Supplementary Fig. 31c and 31d to identify the photodeposited CoOOH. The Co LMM located at 717 eV in the form of metal oxide or oxyhydroxide. c and d do not show distinctly difference, indicating that the CoOOH almost remain the same before and after OWC reaction. It the same for Cr 2p in e and f. Cr peaks at 579.4 eV, in the form of Cr_2O_3 before and after OWS reactions.

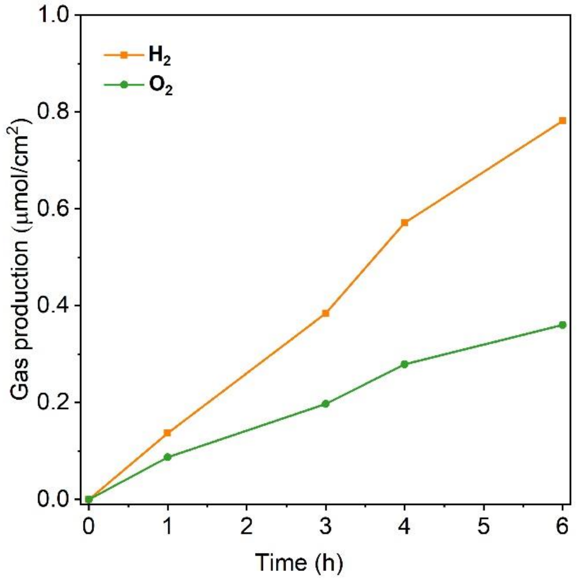

Supplementary Fig. 32 Second overall water splitting reactions of Au array/BTO with cocatalysts in pure water. The activity reduced to almost half that of the first one in Fig. 4d. This may be the result of the Rh^0 is transformed to Rh^{3+} (Supplementary Fig. 31 and 31b).

Supplementary References:

1. Kawano H. Effective work functions for ionic and electronic emissions from mono- and polycrystalline surfaces. *Prog. Surf. Sci.* **83**, 1-165 (2008).
2. Kim CH, Bae CD, Ryu KH, Lee BK, Shin HJ. Local Work Function Measurements on Various Inorganic Materials Using Kelvin Probe Force Spectroscopy. *Solid State Phenom.* **124-126**, 607 (2007).
3. Palermo V*, et al.* Influence of Molecular Order on the Local Work Function of Nanographene Architectures: A Kelvin-Probe Force Microscopy Study. *ChemPhysChem* **6**, 2371-2375 (2005).
4. Böhmisch M, Burmeister F, Rettenberger A, Zimmermann J, Boneberg J, Leiderer P. Atomic Force Microscope Based Kelvin Probe Measurements: Application to an Electrochemical Reaction. *J. Phys. Chem. B* **101**, 10162-10165 (1997).
5. Takata T*, et al.* Photocatalytic water splitting with a quantum efficiency of almost unity. *Nature* **581**, 411-414 (2020).
6. Lee C, Lee YK, Park Y, Park JY. Polarization Effect of Hot Electrons in Tandem-Structured Plasmonic Nanodiode. *ACS Photonics* **5**, 3499-3506 (2018).
7. Gao Y*, et al.* The Polarization Effect in Surface-Plasmon-Induced Photocatalysis on Au/TiO_2 Nanoparticles. *Angew. Chem. Int. Ed.* **59**, 18218 (2020).
8. Spanier JE*, et al.* Power conversion efficiency exceeding the Shockley–Queisser limit in a ferroelectric insulator. *Nat. Photon.* **10**, 611 (2016).
9. Werner JH, Brendel R, Queisser HJ. Radiative efficiency limit of terrestrial solar cells with internal carrier multiplication. *Appl. Phys. Lett.* **67**, 1028-1030 (1995).
10. Liu Q*, et al.* Rules for Selecting Metal Cocatalyst Based on Charge Transfer and Separation Efficiency between ZnO Nanoparticles and Noble Metal Cocatalyst Ag/ Au/ Pt. *ChemCatChem* **12**, 3838-3842 (2020).
11. Wu K, Rodriguez-Cordoba WE, Yang Y, Lian T. Plasmon-induced hot electron transfer from the Au tip to CdS rod in CdS-Au nanoheterostructures. *Nano Lett.* **13**, 5255-5263 (2013).
12. Furube A, Du L, Hara K, Katoh R, Tachiya M. Ultrafast Plasmon-Induced

Electron Transfer from Gold Nanodots into TiO_2 Nanoparticles. *J. Am. Chem. Soc.* **129**, 14852-14853 (2007).

13. Chen F, Klein A. Polarization dependence of Schottky barrier heights at interfaces of ferroelectrics determined by photoelectron spectroscopy. *Phys. Rev. B* **86**, (2012).
14. Choi M, Oba F, Tanaka I. Electronic and structural properties of the oxygen vacancy in $BaTiO_3$. *Appl. Phys. Lett.* **98**, 172901 (2011).
15. Deng G, Li G, Ding A, Yin Q. Evidence for oxygen vacancy inducing spontaneous normal-relaxor transition in complex perovskite ferroelectrics. *Appl. Phys. Lett.* **87**, 192905 (2005).
16. Hashimoto S, Sugie T, Zhang Z, Yamashita K, Noda M. Effects of final annealing in oxygen on characteristics of $BaTiO_3$ thin films for resistance random access memory. *Jpn. J. Appl. Phys.* **54**, (2015).
17. Jia M-X, *et al.* Ferroelectric polarization-controlled resistive switching in $BaTiO_3$/$SmNiO_3$ epitaxial heterostructures. *Appl. Phys. Lett.* **114**, (2019).
18. Maeda K, *et al.* Characterization of Rh−Cr Mixed-Oxide Nanoparticles Dispersed on $(Ga_{1-x}Zn_x)(N_{1-x}O_x)$ as a Cocatalyst for Visible-Light-Driven Overall Water Splitting. *J. Phys. Chem. B* **110**, 13753-13758 (2006).
19. Camposeco R, Hinojosa-Reyes M, Castillo S, Nava N, Zanella R. Synthesis and characterization of highly dispersed bimetallic Au-Rh nanoparticles supported on titanate nanotubes for CO oxidation reaction at low temperature. *Environ. Sci. Pollut. Res. Int.* **28**, 10734-10748 (2021).
20. Kibis LS, Stadnichenko AI, Koscheev SV, Zaikovskii VI, Boronin AI. XPS Study of Nanostructured Rhodium Oxide Film Comprising Rh^{4+} Species. *J. Phys. Chem. C* **120**, 19142-19150 (2016).
