# Supplementary figures and images for: Bipolar charge collecting structure enables overall water splitting on ferroelectric photocatalysts
Source: Nat Commun. 2022 Jul 22;13:4245. doi: 10.1038/s41467-022-32002-y (PMC9307613; doi:10.1038/s41467-022-32002-y)

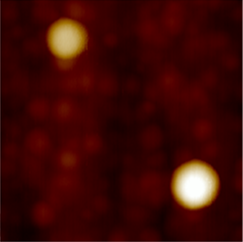

Supplement: Supplementary file 2 — Source Data [file 41467_2022_32002_MOESM2_ESM.zip › SOURCE DATA/1/1b.png]

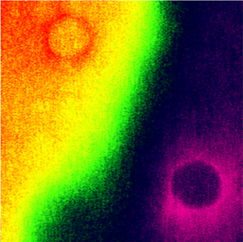

Supplement: Supplementary file 2 — Source Data [file 41467_2022_32002_MOESM2_ESM.zip › SOURCE DATA/1/1c.png]

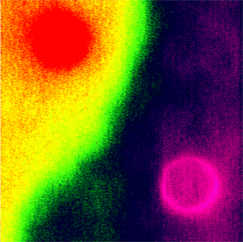

Supplement: Supplementary file 2 — Source Data [file 41467_2022_32002_MOESM2_ESM.zip › SOURCE DATA/1/1d.png]

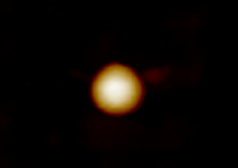

Supplement: Supplementary file 2 — Source Data [file 41467_2022_32002_MOESM2_ESM.zip › SOURCE DATA/3/3a-1.png]

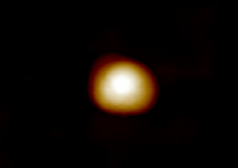

Supplement: Supplementary file 2 — Source Data [file 41467_2022_32002_MOESM2_ESM.zip › SOURCE DATA/3/3a-2.png]

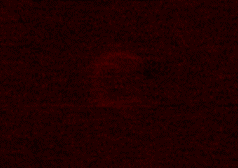

Supplement: Supplementary file 2 — Source Data [file 41467_2022_32002_MOESM2_ESM.zip › SOURCE DATA/3/3b-1.png]

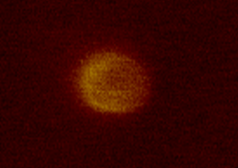

Supplement: Supplementary file 2 — Source Data [file 41467_2022_32002_MOESM2_ESM.zip › SOURCE DATA/3/3b-2.png]

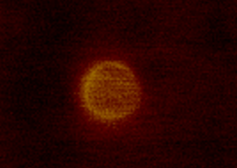

Supplement: Supplementary file 2 — Source Data [file 41467_2022_32002_MOESM2_ESM.zip › SOURCE DATA/3/3c-1.png]

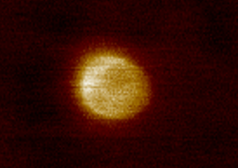

Supplement: Supplementary file 2 — Source Data [file 41467_2022_32002_MOESM2_ESM.zip › SOURCE DATA/3/3c-2.png]

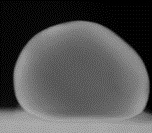

Supplement: Supplementary file 2 — Source Data [file 41467_2022_32002_MOESM2_ESM.zip › SOURCE DATA/3/3d-1.png]

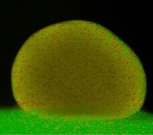

Supplement: Supplementary file 2 — Source Data [file 41467_2022_32002_MOESM2_ESM.zip › SOURCE DATA/3/3d-2.png]

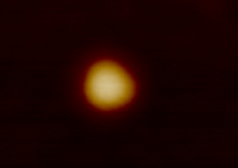

Supplement: Supplementary file 2 — Source Data [file 41467_2022_32002_MOESM2_ESM.zip › SOURCE DATA/3/3e-1.png]

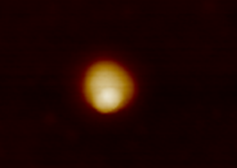

Supplement: Supplementary file 2 — Source Data [file 41467_2022_32002_MOESM2_ESM.zip › SOURCE DATA/3/3e-2.png]

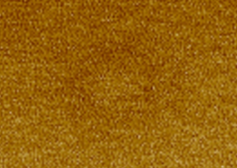

Supplement: Supplementary file 2 — Source Data [file 41467_2022_32002_MOESM2_ESM.zip › SOURCE DATA/3/3f-1.png]

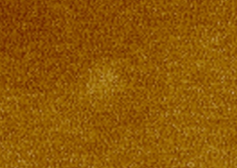

Supplement: Supplementary file 2 — Source Data [file 41467_2022_32002_MOESM2_ESM.zip › SOURCE DATA/3/3f-2.png]

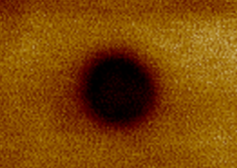

Supplement: Supplementary file 2 — Source Data [file 41467_2022_32002_MOESM2_ESM.zip › SOURCE DATA/3/3g-1.png]

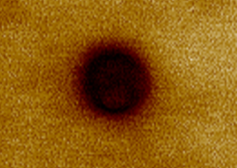

Supplement: Supplementary file 2 — Source Data [file 41467_2022_32002_MOESM2_ESM.zip › SOURCE DATA/3/3g-2.png]

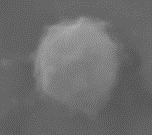

Supplement: Supplementary file 2 — Source Data [file 41467_2022_32002_MOESM2_ESM.zip › SOURCE DATA/3/3h-1.png]

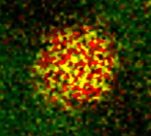

Supplement: Supplementary file 2 — Source Data [file 41467_2022_32002_MOESM2_ESM.zip › SOURCE DATA/3/3h-2.png]

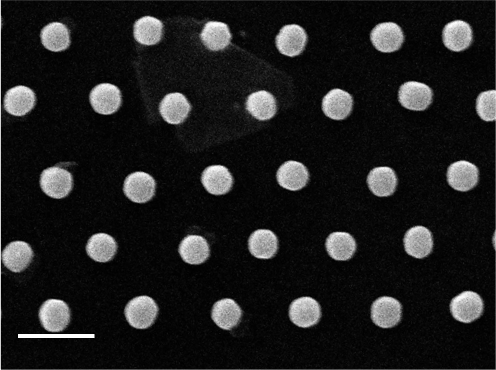

Supplement: Supplementary file 2 — Source Data [file 41467_2022_32002_MOESM2_ESM.zip › SOURCE DATA/4/4a-1.png]

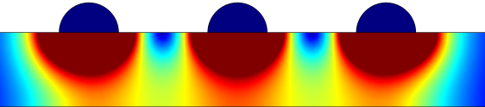

Supplement: Supplementary file 2 — Source Data [file 41467_2022_32002_MOESM2_ESM.zip › SOURCE DATA/4/4a-2.png]

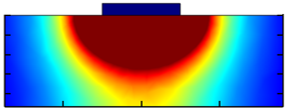

Supplement: Supplementary file 2 — Source Data [file 41467_2022_32002_MOESM2_ESM.zip › SOURCE DATA/S10/S10a.png]

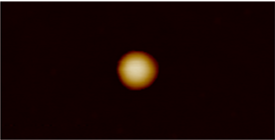

Supplement: Supplementary file 2 — Source Data [file 41467_2022_32002_MOESM2_ESM.zip › SOURCE DATA/S11/S11a.png]

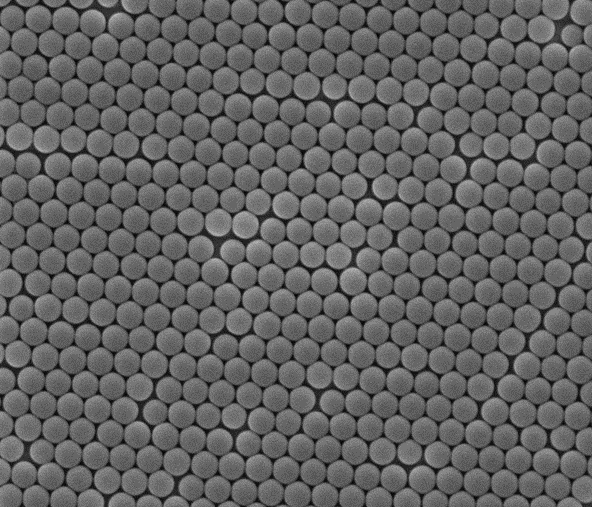

Supplement: Supplementary file 2 — Source Data [file 41467_2022_32002_MOESM2_ESM.zip › SOURCE DATA/S13/S13a.png]

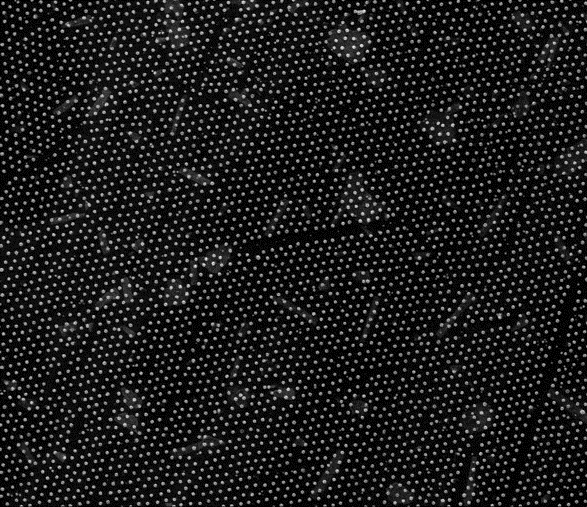

Supplement: Supplementary file 2 — Source Data [file 41467_2022_32002_MOESM2_ESM.zip › SOURCE DATA/S13/S13b.png]

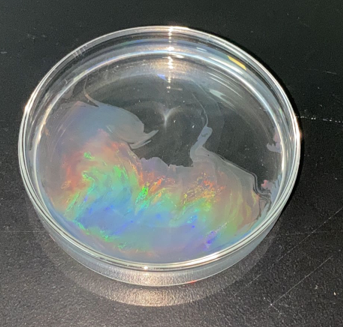

Supplement: Supplementary file 2 — Source Data [file 41467_2022_32002_MOESM2_ESM.zip › SOURCE DATA/S14/S14a.png]

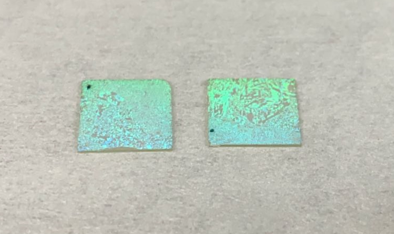

Supplement: Supplementary file 2 — Source Data [file 41467_2022_32002_MOESM2_ESM.zip › SOURCE DATA/S14/S14b.png]

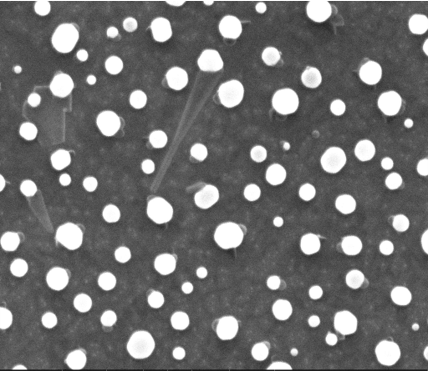

Supplement: Supplementary file 2 — Source Data [file 41467_2022_32002_MOESM2_ESM.zip › SOURCE DATA/S15/S15.png]

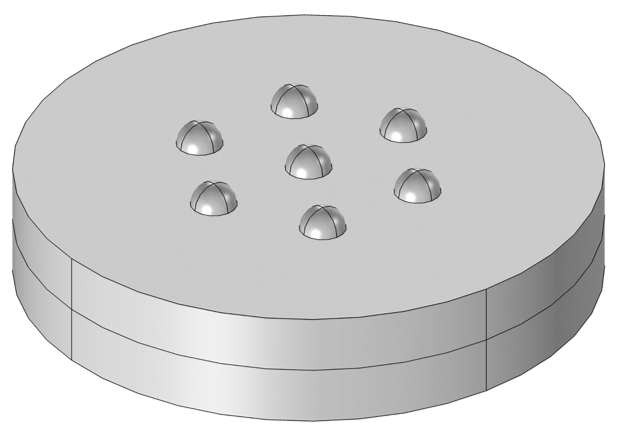

Supplement: Supplementary file 2 — Source Data [file 41467_2022_32002_MOESM2_ESM.zip › SOURCE DATA/S17/S17a.png]

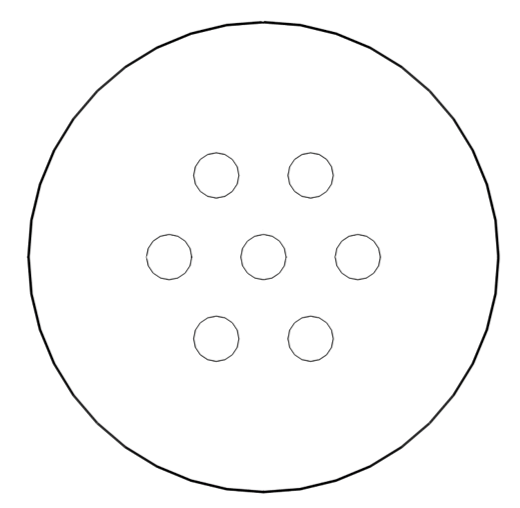

Supplement: Supplementary file 2 — Source Data [file 41467_2022_32002_MOESM2_ESM.zip › SOURCE DATA/S17/S17b.png]

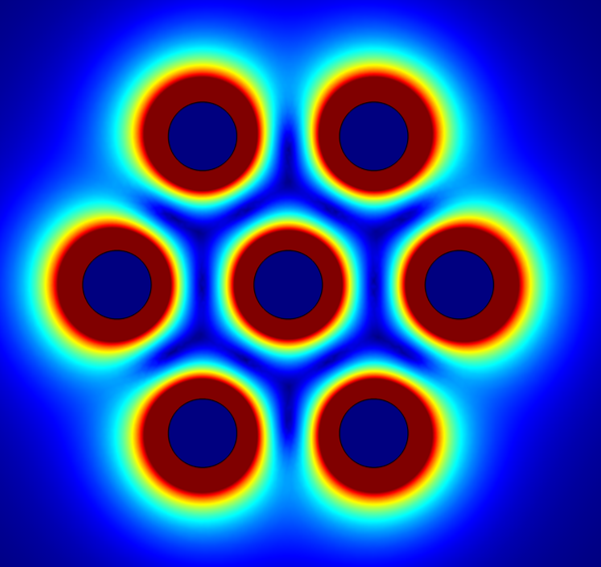

Supplement: Supplementary file 2 — Source Data [file 41467_2022_32002_MOESM2_ESM.zip › SOURCE DATA/S18/S18a.png]

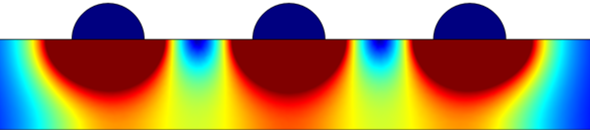

Supplement: Supplementary file 2 — Source Data [file 41467_2022_32002_MOESM2_ESM.zip › SOURCE DATA/S18/S18b-1.png]

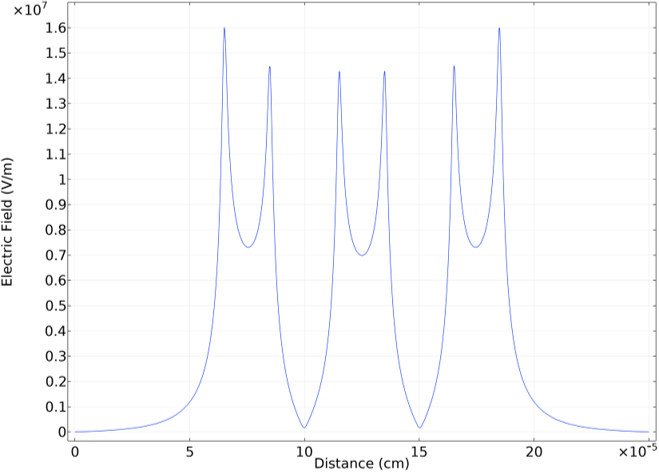

Supplement: Supplementary file 2 — Source Data [file 41467_2022_32002_MOESM2_ESM.zip › SOURCE DATA/S18/S18b-2.png]

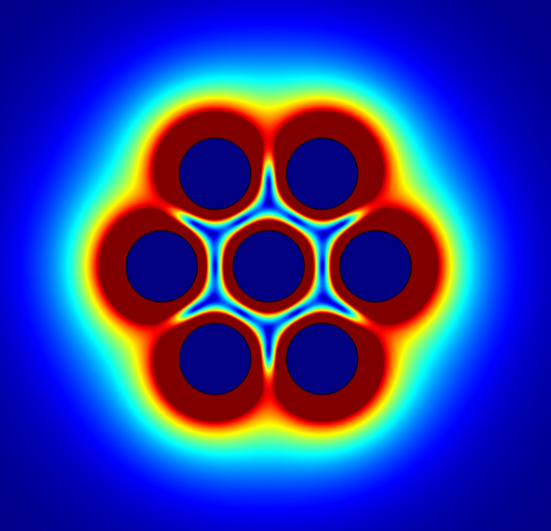

Supplement: Supplementary file 2 — Source Data [file 41467_2022_32002_MOESM2_ESM.zip › SOURCE DATA/S19/S19a.png]

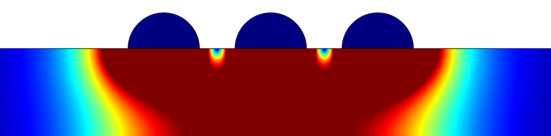

Supplement: Supplementary file 2 — Source Data [file 41467_2022_32002_MOESM2_ESM.zip › SOURCE DATA/S19/S19b-1.png]

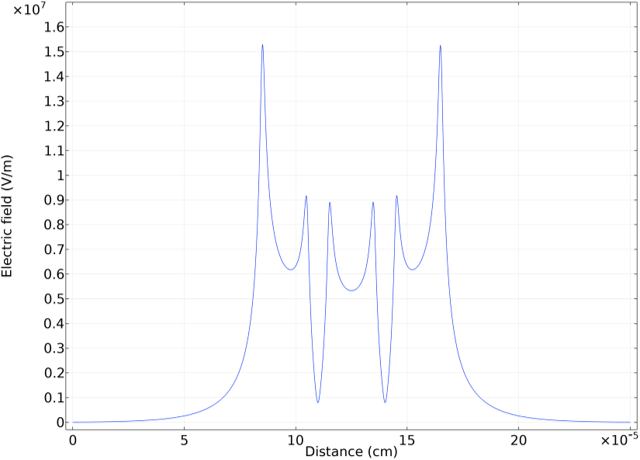

Supplement: Supplementary file 2 — Source Data [file 41467_2022_32002_MOESM2_ESM.zip › SOURCE DATA/S19/S19b-2.png]

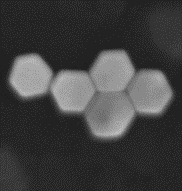

Supplement: Supplementary file 2 — Source Data [file 41467_2022_32002_MOESM2_ESM.zip › SOURCE DATA/S1/S1e.png]

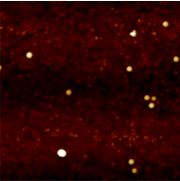

Supplement: Supplementary file 2 — Source Data [file 41467_2022_32002_MOESM2_ESM.zip › SOURCE DATA/S1/S1f.png]

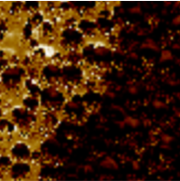

Supplement: Supplementary file 2 — Source Data [file 41467_2022_32002_MOESM2_ESM.zip › SOURCE DATA/S1/S1g.png]

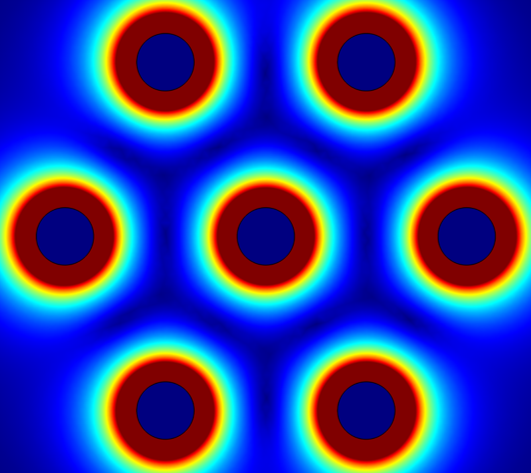

Supplement: Supplementary file 2 — Source Data [file 41467_2022_32002_MOESM2_ESM.zip › SOURCE DATA/S20/S20a.png]

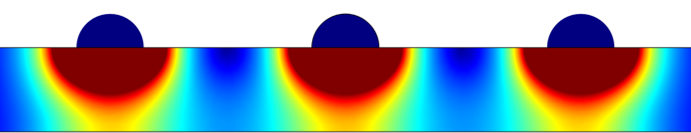

Supplement: Supplementary file 2 — Source Data [file 41467_2022_32002_MOESM2_ESM.zip › SOURCE DATA/S20/S20b-1.png]

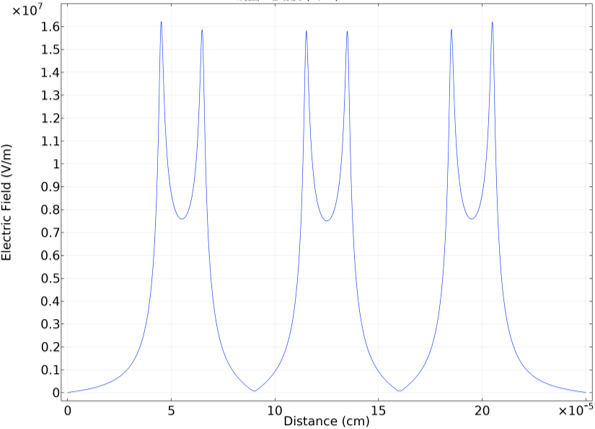

Supplement: Supplementary file 2 — Source Data [file 41467_2022_32002_MOESM2_ESM.zip › SOURCE DATA/S20/S20b-2.png]

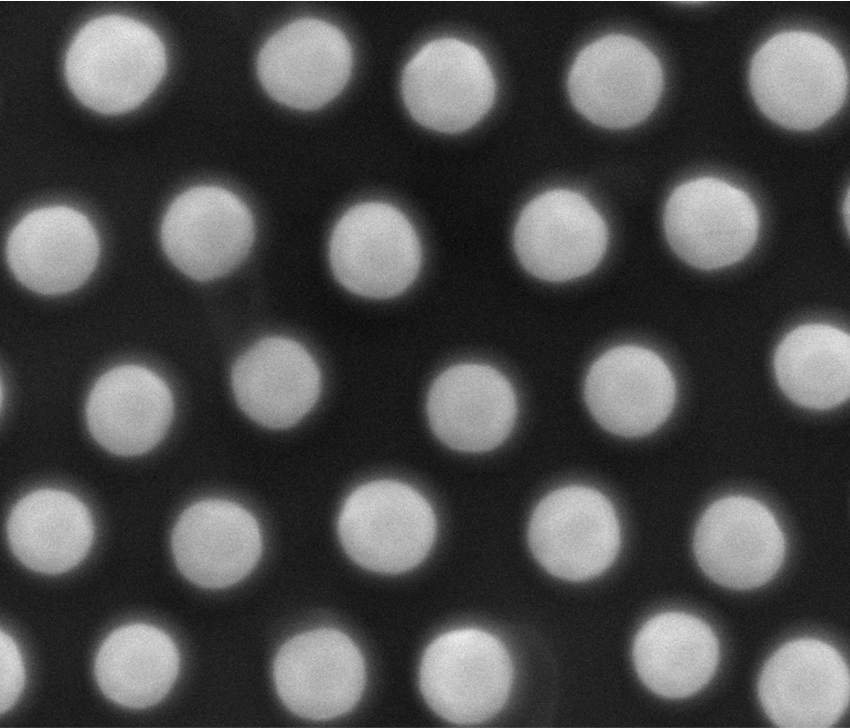

Supplement: Supplementary file 2 — Source Data [file 41467_2022_32002_MOESM2_ESM.zip › SOURCE DATA/S22/S22a.png]

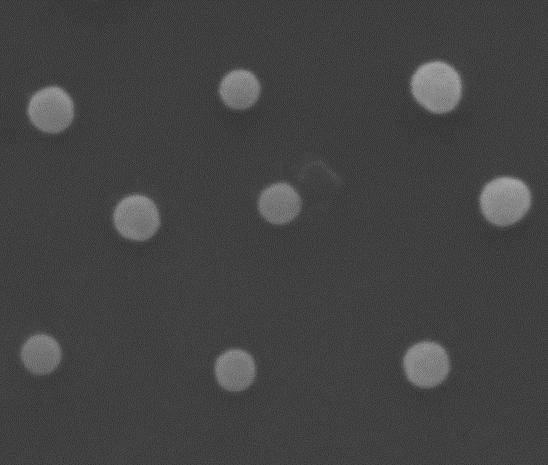

Supplement: Supplementary file 2 — Source Data [file 41467_2022_32002_MOESM2_ESM.zip › SOURCE DATA/S22/S22b.png]

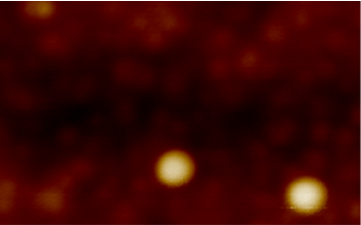

Supplement: Supplementary file 2 — Source Data [file 41467_2022_32002_MOESM2_ESM.zip › SOURCE DATA/S26/S26a.png]

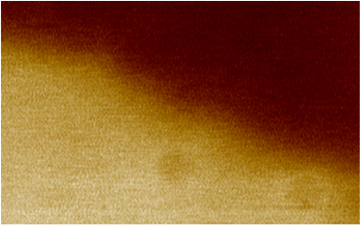

Supplement: Supplementary file 2 — Source Data [file 41467_2022_32002_MOESM2_ESM.zip › SOURCE DATA/S26/S26b.png]

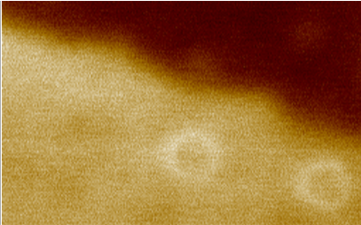

Supplement: Supplementary file 2 — Source Data [file 41467_2022_32002_MOESM2_ESM.zip › SOURCE DATA/S26/S26c.png]

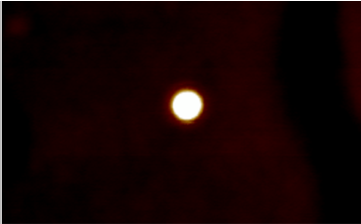

Supplement: Supplementary file 2 — Source Data [file 41467_2022_32002_MOESM2_ESM.zip › SOURCE DATA/S27/S27a.png]

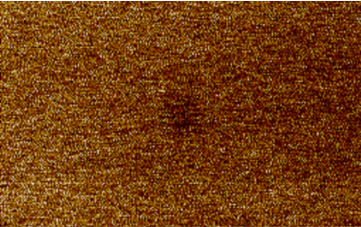

Supplement: Supplementary file 2 — Source Data [file 41467_2022_32002_MOESM2_ESM.zip › SOURCE DATA/S27/S27b.png]

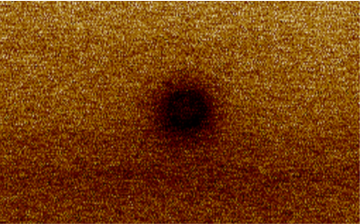

Supplement: Supplementary file 2 — Source Data [file 41467_2022_32002_MOESM2_ESM.zip › SOURCE DATA/S27/S27c.png]

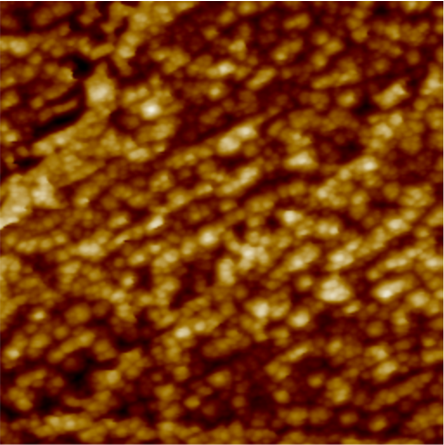

Supplement: Supplementary file 2 — Source Data [file 41467_2022_32002_MOESM2_ESM.zip › SOURCE DATA/S28/S28a.png]

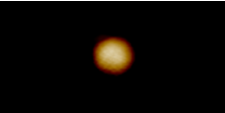

Supplement: Supplementary file 2 — Source Data [file 41467_2022_32002_MOESM2_ESM.zip › SOURCE DATA/S3/S3a.png]

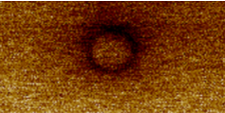

Supplement: Supplementary file 2 — Source Data [file 41467_2022_32002_MOESM2_ESM.zip › SOURCE DATA/S3/S3b.png]

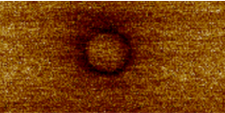

Supplement: Supplementary file 2 — Source Data [file 41467_2022_32002_MOESM2_ESM.zip › SOURCE DATA/S3/S3c.png]

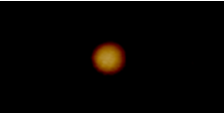

Supplement: Supplementary file 2 — Source Data [file 41467_2022_32002_MOESM2_ESM.zip › SOURCE DATA/S3/S3d.png]

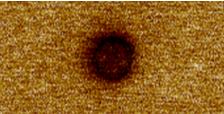

Supplement: Supplementary file 2 — Source Data [file 41467_2022_32002_MOESM2_ESM.zip › SOURCE DATA/S3/S3e.png]

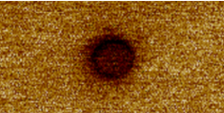

Supplement: Supplementary file 2 — Source Data [file 41467_2022_32002_MOESM2_ESM.zip › SOURCE DATA/S3/S3f.png]

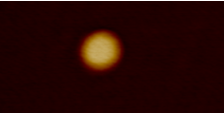

Supplement: Supplementary file 2 — Source Data [file 41467_2022_32002_MOESM2_ESM.zip › SOURCE DATA/S4/S4a.png]

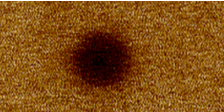

Supplement: Supplementary file 2 — Source Data [file 41467_2022_32002_MOESM2_ESM.zip › SOURCE DATA/S4/S4b.png]

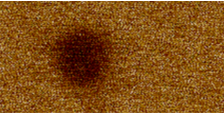

Supplement: Supplementary file 2 — Source Data [file 41467_2022_32002_MOESM2_ESM.zip › SOURCE DATA/S4/S4c.png]

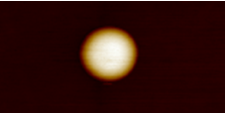

Supplement: Supplementary file 2 — Source Data [file 41467_2022_32002_MOESM2_ESM.zip › SOURCE DATA/S5/S5a.png]

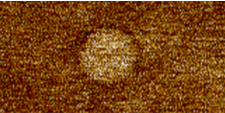

Supplement: Supplementary file 2 — Source Data [file 41467_2022_32002_MOESM2_ESM.zip › SOURCE DATA/S5/S5b.png]

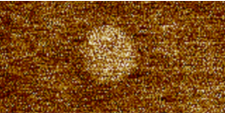

Supplement: Supplementary file 2 — Source Data [file 41467_2022_32002_MOESM2_ESM.zip › SOURCE DATA/S5/S5c.png]

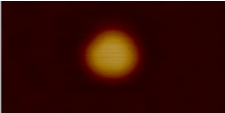

Supplement: Supplementary file 2 — Source Data [file 41467_2022_32002_MOESM2_ESM.zip › SOURCE DATA/S6/S6a.png]

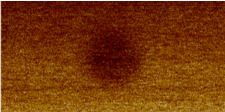

Supplement: Supplementary file 2 — Source Data [file 41467_2022_32002_MOESM2_ESM.zip › SOURCE DATA/S6/S6b.png]

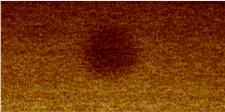

Supplement: Supplementary file 2 — Source Data [file 41467_2022_32002_MOESM2_ESM.zip › SOURCE DATA/S6/S6c.png]

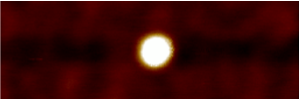

Supplement: Supplementary file 2 — Source Data [file 41467_2022_32002_MOESM2_ESM.zip › SOURCE DATA/S7/S7a.png]

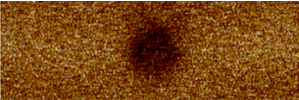

Supplement: Supplementary file 2 — Source Data [file 41467_2022_32002_MOESM2_ESM.zip › SOURCE DATA/S7/S7b.png]

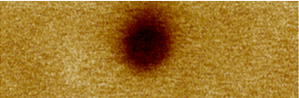

Supplement: Supplementary file 2 — Source Data [file 41467_2022_32002_MOESM2_ESM.zip › SOURCE DATA/S7/S7c.png]

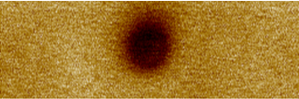

Supplement: Supplementary file 2 — Source Data [file 41467_2022_32002_MOESM2_ESM.zip › SOURCE DATA/S7/S7d.png]
